# An Integrative Multi-Omics Analysis of The Molecular Links between Aging and Aggressiveness in Thyroid Cancers

**DOI:** 10.14336/AD.2022.1021

**Published:** 2023-06-01

**Authors:** Emmanuelle Ruiz, Emad Kandil, Solomon Alhassan, Eman Toraih, Youssef Errami, Zakaria Y. Abd Elmageed, Mourad Zerfaoui

**Affiliations:** ^1^Louisiana State University, School of Veterinary Medicine, Baton Rouge, LA 70803, USA.; ^2^Department of Surgery, Tulane University School of Medicine, LA 70112, USA.; ^3^Department of Pharmacology, Edward Via College of Osteopathic Medicine, University of Louisiana, Monroe, LA 71203.

**Keywords:** aging, aggressiveness, thyroid cancer, risk stratification, miRNAs, poor prognosis

## Abstract

Aging modifies risk in all cancers, but age is used as a clinical staging criterion uniquely in thyroid cancer (TC). The molecular drivers of age-dependent TC onset and aggressiveness remain poorly understood. We applied an integrative, multi-omics data analysis approach to characterize these signatures. Our analysis reveals that aging, independent of BRAF^V600E^ mutational status, drives a significant accumulation of aggressiveness-related markers and poorer survival outcomes, most noticeably at age 55 and over. We identified that chromosomal alterations in loci 1p/1q as aging-associated drivers of aggressiveness, and that depleted infiltration with tumor surveillant CD8+T and follicular helper T cells, dysregulation of proteostasis- and senescence-related processes, and ERK1/2 signaling cascade are key features of the aging thyroid and TC onset/progression and aggressiveness in aging patients but not in young individuals. A panel of 23 genes, including those related to cell division such as CENPF, ERCC6L, and the kinases MELK and NEK2, were identified and rigorously characterized as aging-dependent and aggressiveness-specific markers. These genes effectively stratified patients into aggressive clusters with distinct phenotypic enrichment and genomic/transcriptomic profiles. This panel also showed excellent performance in predicting metastasis stage, BRAF^V600E^, TERT promoter mutation, and survival outcomes and was superior to the American Thyroid Association (ATA) methodology in predicting aggressiveness risk. Our analysis established clinically relevant biomarkers for TC aggressiveness factoring in aging as an important component.

## Introduction

Aging is considered a risk factor for a wide array of chronic malignancies. Even when race, sex, diet, and other variables are controlled for, clear disparities in cancer onset, prognosis, and clinical outcomes among different age categories have been well established [1]. Many features of the aging process (genomic instability, telomere loss, dysregulation of proteostasis, etc.) drive tumorigenesis/cancer progression or at least contribute to a pro-oncogenic cellular landscape [2]. Clinical evidence shows that aging in thyroid cancer (TC) is associated with tumor size and stage, lymph node metastasis (LNM), distant metastasis stage, extrathyroidal extension (ETE), and histological type. Thus, elucidating the cellular and molecular basis for the impact of age on cancer is an active area of research [3, 4]. According to the cancer prognosis and staging guidelines established by the American Joint Committee on Cancer (AJCC), TC is the only cancer for which patient age is regarded as the principal prognosis/staging criterion, with the most recent guidelines (8^th^ edition) using age 55 as staging cut-off [5]. This cut-off is also used as a predictor of aggressiveness and an AJCC staging classifier of overall survival (OS) outcome of thyroid patients. Patients younger than 55 are grouped into stages I or II, differentiated by the presence of distant metastasis. Patients older than 55 are stratified into stages I, II, III, or IV, according to tumor size, ETE, and the presence of regional and distant metastasis [6]. There remains some debate regarding this cut-off age (previous AJCC staging guidelines recommended an age of 45 [7-10]), but 55 is thought to increase accuracy of staging and overall outcome prediction [11-16] for differentiated TCs.

Studies on other cancer types have highlighted important differences in the mutational profile of early- and late-age onset tumors. Distinct oncogenic processes and pathways are known to drive induction and aggressiveness in young and old individuals [3, 17]. For several cancer types, a clear age-dependent shift has been shown in hormone sensitivity, the expression of key regulatory genes (oncogenes, transcription factors [TFs], kinases, microRNAs, etc.), driver mutation burden, systemic/peritumoral immunity, DNA methylation, and genomic instability, and these factors are significantly associated with aggressiveness [18]. The development and clinical adoption of high-throughput sequencing technologies and the public availability of multi-omics datasets create opportunities for multidimensional profiling of the molecular landscape and drivers of TC aggressiveness. In this study, we carried out a deep integrative multi-omics analysis to uncover molecular signatures and pathways unique and common to young and aging TC patients. First, we characterized the age-dependent distribution of TC aggressive tumor phenotypes and examined the validity of AJCC’s recommended age cut-off for staging and stratification of TC risk. We identified and functionally characterized the genomic, transcriptomic, and immune cell infiltration profiles of tumors, linking molecular signatures to relevant phenotypes and survival outcomes in different age categories. A rigorous functional analysis of signatures was carried out to identify critical pathways and processes that mediate the influence of aging on TC. Our analysis allowed the identification and validation of potentially useful prognostic markers for aging TC and the stratification of patients according to their aggressiveness risks and molecular features.

## METHODS

### Data

The Genotype Tissue Expression files for the gene expression dataset and sample annotation information (“GTEx_Analysis_2017-06-05_v8_RNASeQCv1.1.9_ gene_tpm” and “GTEx_Analysis_v8_Annotations_ SampleAttributesDD.xlsx,” respectively) were retrieved from the GTEx portal. For this study, 653 thyroid samples were retrieved.

The Cancer Genome Atlas (TCGA) dataset files “Merge_rnaseqv2__illuminahiseq_rnaseqv2__unc_edu__Level_3__RSEM_genes_normalized__data.Level_3.2016012800.0.0.tar.gz,” “Merge_mirnaseq__ illuminahiseq _mirnaseq__bcgsc_ca__Level_3__miR_isoform_expression__data.Level_3.2016012800.0.0.tar.gz,” “Merge_ methylation__humanmethylation450__jhu_usc_edu__Level_3__within_bioassay_data_set_function__data.Level_3.2016012800.0.0.tar.gz,” “Merge_snp__genome_ wide _snp_6__broad_mit_edu__Level_3__segmented_scna_minus_germline_cnv_hg19__seg.Level_3.2016012800.0.0.tar.gz,” “Mutation_Packager_Oncotated_ Calls.Level _3.2016012800.0.0.tar.gz,” and “Merge_Clinical.Level_ 1.2016012800.0.0.tar.gz” were collected through the FireBrowse platform. These files contained data on mRNA and miRNAs expression data, DNA methylation, copy number variation, and clinical information, respectively. For external validation, additional datasets (accession numbers GSE60542, GSE29265, GSE53157, GSE129 879, and GSE65074) were obtained from the Gene Expression Omnibus (GEO).

### Differential expression analyses

Differential Gene Expression (DGE) analysis was performed with the DESeq2 package [19] with the likelihood ratio statistic (LRT) test, adjusting for gender and race in the TCGA cohort or with gender and Hardy score in the GTEx cohort. Differentially expressed mRNA genes (DEGs) and miRNAs (DEmiRNAs) are defined as genes with an adjusted p-value < 0.05 and an absolute value of the log_2_ fold change ≥ 1 between comparisons. The Aging Cohort and Young Cohort are samples from patients aged ≥ 55 and < 55, respectively. Differentially expressed genes/miRNAs that are unique to specific cohorts were identified by filtration steps and comparisons via Venn diagram analysis. Dysregulated genes specific to aging or young cohorts/samples are prefixed by AC (aging cohort) or YC (young cohort).

### Clinical and molecular parameter association analyses

Univariate and multivariate binomial logistic regressions were performed with the glm R function (glm [family = “binomial”]). Multivariate analyses adjusted for gender and race, or for gender, race, LNM, ETE, tumor size, and histological type. Pearson’s chi-square tests (chisq.test function), Kruskal tests (Kruskal.test function), and Spearman correlations (cor.test function) were also used. Only values with adjusted p-value < 0.05 are considered significant in all further downstream analysis.

Survival data were analyzed with a Cox regression model and likelihood ratio test, and plotted with as Kaplan-Meier curve (survminer [20] and survival [21] R packages, p-value estimated with the log-rank test). There were no death events in the young cohort, so OS and disease-specific survival (DSS) were not systematically analyzed with a Cox model, since it would influence the regression, leading to a false hazard ratio (HR) estimation and a false Wald p-value.

To estimate the prognostic potential of the identified gene panel, a prediction model was designed with a linear discriminant analysis (LDA) regression performed with LDA R function (MASS [22] R package) for each clinical parameter analyzed and a Cox regression model for survival data. The prediction values (computed with the predict () function in R) were used for the estimation of sensitivity, specificity, positive predictive value (PPV), and negative predictive value (NPV). Receiver Operative Characteristics (ROC) and area under curve (AUC) were estimated with OptimalCutpoints [23] R package. Concordance or C-index [24] and Brier score [25] were calculated with the concordance.index R function (survcomp [26] R package) and BrierScore R function (DescTools [27] R package), respectively. Statistical differences between AUC and C-index were estimated using the DeLong [28] Test (roc.test function - pROC [29] R function) and cindex.comp [30] R function (survcomp R package).

RISK scores were calculated according to the following equation: LDA or Cox beta-coefficient: ∑ xi*βi, with *xi* = expression value of DEG I, and *βi* = regression coefficient of AC-DEGs/YC-DEGs *i* for a specific clinical parameter analysis (from LDA or Cox regression).

### Identification of the aging cancer aggressiveness signature(s)

To identify a signature of mRNA genes/miRNAs that are aging cohort-specific and associated with an aggressive phenotype, we ran DGE analyses with a four-step methodology. Aging-cancer (AC) specific genes/miRNAs were selected based on three criteria. First, genes/miRNAs had to present a significant adjusted p-value and fold change in cancer vs. normal comparison for the aging counterpart cohort (19 cancer vs. 19 normal). Secondly, genes/miRNAs did not have to present a significant adjusted p-value and fold change in the comparison of cancer vs. normal in the younger counterpart cohort (38 cancer vs. 38 normal), or the comparison of old vs. young in the normal cohort (19 old vs. 38 young). Only genes/miRNAs with an absolute value of the log2 fold change > 1 were selected.

To identify genes/miRNAs signatures associated with TC aggressiveness, we adopted a robust methodology for the whole cohort, including young and old tumor samples. First, DEG analysis was performed with binomial clinical parameters, adjusted for gender and race, with the DESeq2 package; continuous variables were stratified as low or high for values lower or higher than their means, respectively. Among the clinical parameters were clinical features/markers (LNM, ETE, tumor size, distant metastasis, histological type, neoplasm focality, primary tumor laterality, etc.); molecular markers (*BRAF* and *TERT* promoter mutation status, differentiation score; survival indicators (disease-free survival [DFS], progression-free interval [PFI], OS, DSS, and disease-free interval [DFI]); and markers related to genomic instability and immunity. Genes/miRNAs had to present an adjusted p-value <0.05 for at least two of the following parameters to be further selected: LNM (N1 vs. N0), ETE (present vs. absent), tumor size (≥1 cm vs. <1cm), and aggressive histological type (T/CPTC vs. FPTC). Hierarchical clustering was performed to cluster the genes/miRNAs according to their transformed adjusted p-value (log2 adjusted p-value with the sign matching the corresponding log2 fold change) for each parameter analyzed. This step was crucial to filter only the genes/miRNAs that were upregulated in the cancer cohort and positively associated with aggressive parameters, or that were downregulated in the cancer cohort and negatively associated with aggressive parameters.

Since an aim of the study was to decipher the TC aggressive phenotype related to aging, a third selection step was performed to collect only the genes/miRNAs that were dependent on aging to be associated with an aggressive phenotype. Hence, an age, gender, and race-adjusted multivariate analysis was also performed. For each parameter analyzed, genes/miRNAs were further selected if they presented an adjusted p-value < 0.05 in the gender/race-adjusted analysis and if they presented an adjusted p-value>0.05 (non-significant) in the age/gender/race-adjusted analysis.

Finally, an internal validation was performed in the 160 aged tumor samples to validate the potential of the AC-DEGs or DE miRNA-AC selected to be associated with an aggressive phenotype in the aging cohort. Gender and race-adjusted multivariate analyses were performed for nine specific parameters reflecting a clinical aggressive phenotype and worse outcome: LNM (N0 vs. N1), ETE (present vs. absent and gross vs. non-gross), histological type (C/TPTC vs. FPTC and TPTC vs. F/CPTC), tumor size (≥1 cm vs. <1cm and ≥2 cm vs. <2cm), differentiation score (low vs. high), *BRAF* and *TERT* promoter mutation status (MUT vs. WT), DFS, PFI, OS, and DSS. A gene was selected if it presented at least one significant association (adjusted p-value < 0.05).

This methodology was chosen after adjusting parameters for each step described previously. Multivariate or univariate analysis was carried out as follows. Step 1: whole cohort, counterpart, or paired cohort analysis; Step 2: genes/miRNAs significantly associated with 0, at least 2, or at least 3 on the 4 parameters proposed; Step 3: hierarchical clustering with all the parameters analyzed or only the clinical/pathological parameters; Step 4: age as a continuous variable or as a binomial variable, aging dependence described if the adjusted p-value was non-significant or superior to the corresponding p-value obtained from the gender and race multivariate analysis. All possibilities were tested, and the final gene signatures’ propensity to predict aging aggressive phenotype and worse outcomes were assessed with Receiver Operator Characteristics area under curve (AUC) estimation for the parameters described in Step 4. For the survival data, AUCs were also compared to those obtained with the ATA risk stratification, and a DeLong test evaluated the significant difference between the two AUCs analyzed. The signature leading to the highest AUC and most significant DeLong p-value was chosen. A similar study was performed in the young cohort, and 65 DEGs were selected.

### Stratification of samples according to the aggressive panel of genes identified

A principal component analysis (PCA) was performed with the 23 DEGs across the aging cohort using the FactoMineR [31] and factoextra [32] R packages. Next, a PCA aging score was calculated according to the equation:

$$W=\sum \left(\left|L\mathrm{ij}\right|\mathrm{*Ei}\right)\mathrm{PCA Aging score}=\frac{\sum Xi\mathrm{*Wi}}{\sum Wi}$$

with *L_ij_*, loading value of the *i*^th^ variable of grouping on *j*^th^ PC, *E_i_*, eigenvalue of the *j*^th^ PC, *W_i_*, weight of the *i*^th^ variable, *X_i_*, the normalized value of *i*^th^ variable. Only PCs with E > 1 were selected.

A score > 0 characterized samples with a more aggressive phenotype (MAAC = more aggressive aging cluster) and a score < 0 characterized samples with a less aggressive phenotype (LAAC = less aggressive aging cluster). A similar analysis was performed for the young cohort from the 65 aggressiveness-related genes, stratifying young tumor samples into a more aggressive young cluster (MAYC) and a less aggressive young cluster (LAYC).

### Immune infiltration percentage estimation

Immune infiltration percentages were estimated using the CIBERSORT algorithm [33] with the 23-gene signature, identifying 22 human hematopoietic cell phenotypes.

### KEGG pathways and GO entries enrichment analyses

Pathway enrichment analyses were performed with pathfindR package [34] using the active-subnetwork-oriented enrichment method. For the TCGA and GTEx cohort analyses, DEGs were used as input. Next, for each sample, an agglomerate z-score was calculated for the enriched pathways identified. Using the agglomerate z-scores, logistic regressions were performed to select only the pathways significantly associated with the condition analyzed (adjusted p-value < 0.05).

Each aging comparison was meticulously evaluated by extracting only the pathways that were specific to aging. Similar analyses were performed in the younger cohort, and pathways that were common in both cohorts (with the same direction of enrichment) and were not significantly different (with a Kruskal test p-value) were removed from the list of “aging-related” pathways. Pathways were clustered following a PCA analysis computing the agglomerate z-scores across the samples analyzed.

To summarize the enrichment pathway analyses, normalized enrichment scores (NES) for each pathway were calculated in all samples (in the normal, aging, and young cohorts). Briefly, the list of genes included in each entry analyzed was gathered with KEGGREST [35] and biomaRt [36] R packages. With the function ssgsea () (corto R package [37]), a score was estimated for each input gene list after normalization of their expression. Normalized enrichment scores (NES) were compared using the EdgeR [38] R package to estimate the significant difference observed during aging/young tumor progression and according to the aggressive phenotype hierarchy. Adjusted p-value < 0.05 was considered significant.

**Figure 1.**
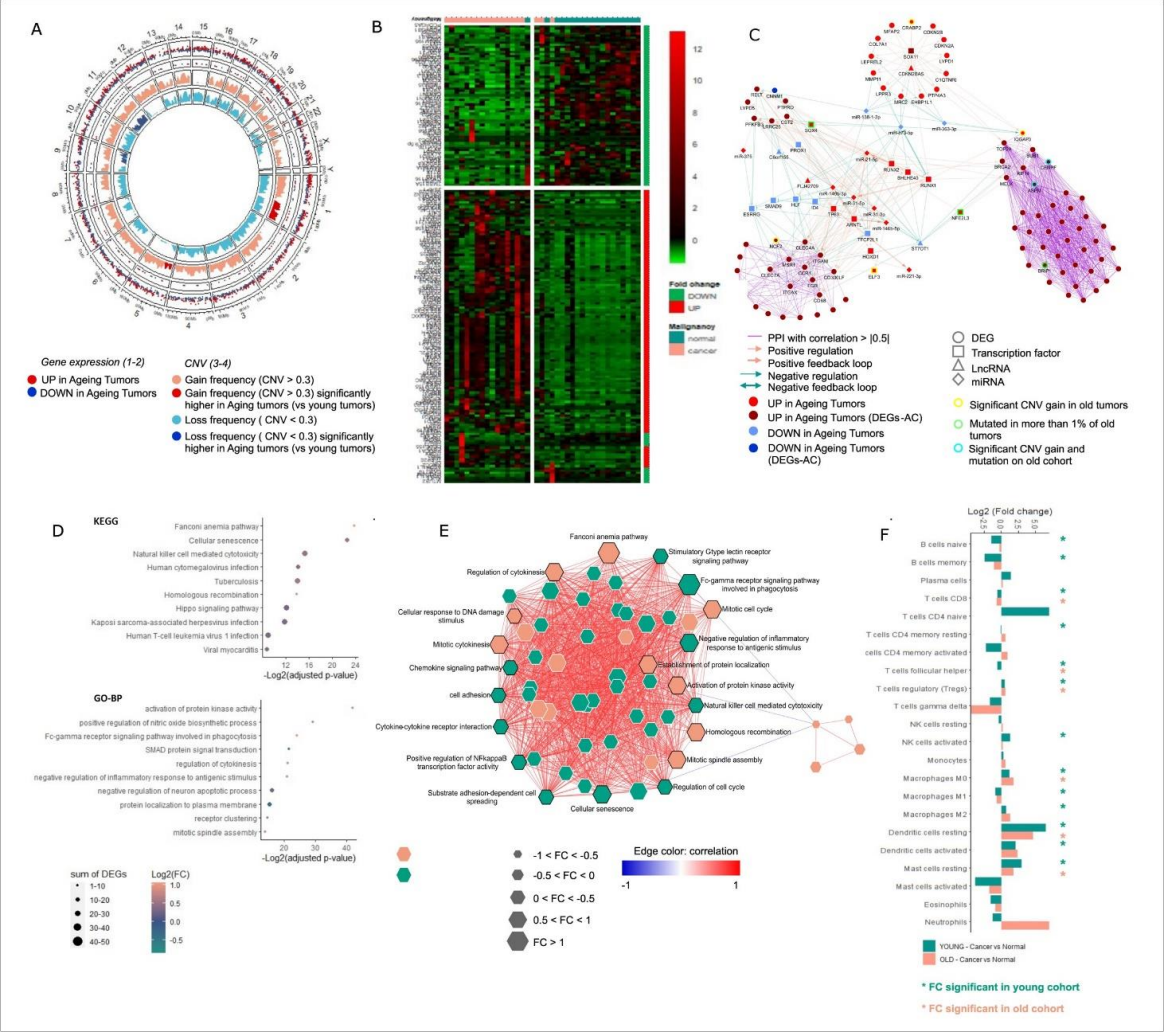
Aging-induced thyroid transformation landscape. (A) Circular plot representing (from outer to inner) (1) Log2 fold change of the DEG/miRNAs expression between tumor vs normal counterpart samples, (2) the Aging-Cancer specific DEG/miRNAs-AC, and the frequency of gene copy number (3) gain (CNV > 0.3) or (4) loss (CNV < -0.3) in aging cancer cohort. Similar analysis for the young cohort comparison is plotted in Supplementary Fig. 5. (B) Heatmap representing a hierarchical clustering of ageing tumors and normal paired samples according to the aging-cancer specific 198 DEG/miRNAs (AC-DEGs). (C) PPI and regulatory network of TFs, miRNAs and LncRNAs strongly controlling the 198 AC- DEG/miRNAs. Only TF/miRNA/DEG, TF/LncRNA/DEG, or miRNA/LncRNA/DEG loops and PPI edges with spearman correlation > |0.5|, unique to the aging cohort, were selected. (D) Dot plots representing the top ten most significant KEGG (top) and GO-BP (bottom) pathways selected after an enrichment analysis, according to their fold change, corresponding adjusted p-value, and their number of DEGs involved. Only pathways involving AC-DEGs and unique tumor-aging or significantly different from the younger comparison were finally selected. (E) Network representing the spearman correlation between the enriched pathways in the aging cohort, with the node size depending on the pathway agglomerate z-score’ fold change (Old cancer vs Old normal samples). Two clusters were identified through a PCA computing the pathways’ agglomerate scores among the samples. Only the KEGG and GO-BP entries with the highest contribution to the first two PCA were labeled. (F) Bar plot representing immune cell infiltration mean in the young and old samples cohorts. * Adjusted p-value < 0.05. CNV, Copy number variation, FC, Fold change. PPI, Protein-protein interaction; TF, Transcription factor.

### Protein-protein interaction (PPI) and regulatory networks

Protein-protein interaction (PPI) data, long non-coding RNA (lncRNA) interactions, and TFs regulations were collected with the STRING [39] database, the RNAInter [40] database, and “tftargets.rda” file (R Curl R package including the Marbach2016, ENCODE, and ITFP databases https://github.com/slowkow/tftargets), respectively. miRNA interaction prediction was performed on 3’UTR, CDS, 5’UTR, and promoter with the MirWalk.2.0 [41] platform. Spearman correlations were calculated, and only correlations greater than |0.5| were selected. Loops between miRNA/TF/DEG, miRNA/LncRNA/DEG, and TF/LncRNA/DEG were identified and plotted in networks. “Inhibition” and “activation” were characterized according to the correlation between regulators and DEGs. miRNAs were considered activators if interactions on the promoter were identified with a positive correlation. Conversely, miRNAs were considered inhibitors if an interaction on 3’UTR, CDS, or 5’UTR was described with a negative correlation.

### Copy number variation (CNV) analysis

CNVs were calculated with GISTIC.2.0 [42]. Alterations were identified as a gain with a CNV > 0.3 and as a loss with CNV < -0.3. The frequency of loss and gain were then calculated for each group of samples studied and expressed as the ratio of the number of samples presenting a gain or a loss to the total number of samples. Frequencies higher than 2% were considered relevant. Pearson’s chi-square tests (with chisq.test R function) were performed to identify which loci and genes were significantly differentially altered across the two groups of samples. In parallel, a Spearman correlation was run between the CNV and the expression of genes. This step was crucial to select genes whose expression is affected by their copy number alteration.

### Plot design

Box plots, forest plots, dot plots, volcano plots, and bar plots were designed with ggplot2 [43] R package. Kaplan-Meier curves were plotted with ggsurvplot (survminer R package). ROC curves were drawn with ggplot2 geom_roc function (plotROC [44] and ggsci [45] R packages). PCA plots (individuals and variables), heatmaps, and circular plots were designed with factoextra, pheatmap [46], and circlize [47] R packages, respectively. Finally, networks were built with Cytoscape 3.8 [48].

## RESULTS

### Age as a prognostic marker for thyroid carcinoma aggressiveness

We stratified the 481 samples into five age groups: < 35, 35-44, 45-54, 55-64, and > 65, to investigate the association between age and aggressiveness phenotypes and validate the selection of age 55 as a prognostically useful cut-off for staging using the TCGA dataset. We then performed logistic regression adjusting for gender and race. With aging, an independent higher risk of aggressive histological subtypes (TPTC vs. C/FPTC, OR=3.0, p=1.1 x 10^-2^), presence of ETE (present vs. absent, OR=2.3, p=2.0 x 10^-3^; gross vs. non-gross, OR=13.3, p=1 x 10^-3^) and the presence of *TERT* promoter mutation (OR=12.5, p=9.1 x 10^-6^) (Supplementary Fig. 1A) was observed. Worse prognosis, as defined by lower DFS, PFI, OS, and DSS, was also observed with aging (Supplementary Fig. 1B, C). Their hazard ratios (HR, probability of event occurrence as death or recurrence) were significantly high (DFS, HR=2.2, p=1.7 x 10^-1^; PFI (HR=2.2, p=4.0 x 10^-3^; OS, p=5.1 x 10^-7^; DSS, p=4.7 x10^-4^) (Supplementary Fig. 1C). A comparison of samples age ≥ 55 vs < 55 also showed a higher risk of key aggressiveness parameters in the aging samples (Supplementary Fig. 1D). We also compared stages and risk categories within AJCC (8^th^ edition) and ATA guidelines in the whole TCGA cohort, stratifying the cohorts by age. As shown in Supplementary Fig. 2, neither staging system presented a significant difference in outcomes between stages/risk categories in either age cohort (< 55 and ≥ 55). This was particularly true comparing survival measures between the AJCC stage II and III and the ATA’s “low” and “intermediate” reoccurrence risk levels. Thus, stratifying by age alone was insufficient in predicting survival and recurrence outcome.

### The molecular landscape of aging-induced tumorigenesis

Changes in the molecular landscape of cells are known to be induced by carcinogenesis and by aging/senescence [2, 49]. To characterize these at the genomic level in TC, we carried out an analysis of CNV to determine age-related structural changes in the genome in TC samples. Using the GISTIC tool, we observed that TCs (independent of age) did not present deep genomic alterations. In 475 samples with CNV information, only 5,369 genes in 167 different loci showed a CNV > 1 (amplification) in 1-6 samples, and 680 genes in 66 different loci showed a CNV less than -1 (loss) in 1-4 samples. Hence, we studied CNVs by age, defining a shallow gain as CNV > 0.3 and a shallow loss as CNV < -0.3. The aging tumor cohort presented specific gains on the 1p, 4p, 5p, 6p, 14q, 19p/q, and 20p/q chromosomal loci and specific loss on the 2p/q, 8p/q, 9p/q, 10p/q, 11p/q, 16q, 17p, 19p, and 21p/q loci (Fig. 1A). Losses on 9p/q, 10p/q and 17p are particularly important, as these chromosomal arms host the tumor suppressor genes CDKN2A, PTEN, and TP53, respectively.

**Figure 2.**
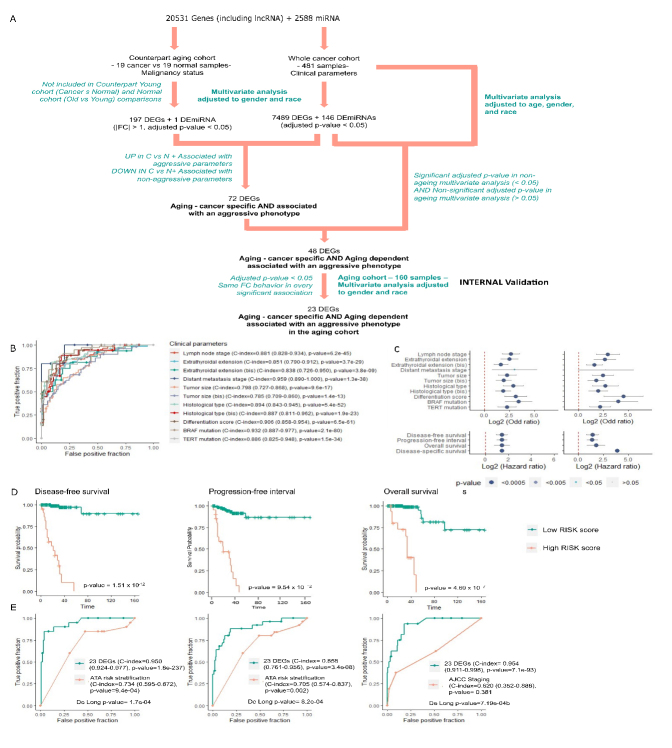
The 23-AC-DEGs signature is prognosis marker for ageing thyroid cancer patients. (A) Workflow for selection of biomarker panel as Ageing-Cancer specific / Ageing dependent aggressive DEGs in the old tumor cohort. (B) Receiver operator characteristics (ROC) curves estimating the accuracy of the 23-AC-DEGs signature prediction for aggressive clinical parameters. (C). Logistic regression analyses with the 23-AC-DEGs risk score with aggressive clinical parameters, in a univariate and multivariate (adjusted to gender and race) analyses. (D). Kaplan-Meier curves showing probability of survival according to the 23 AC-DEGs derived risk score dependent stratification of samples for disease-free survival (left), progression-free interval (middle), and overall survival (right). Log-rank p-value significant < 0.05. (E) Receiver operator characteristics (ROC) curves for the accuracy of the 23-AC-DEGs signature prediction for outcome parameters, comparing with ATA risk stratification prediction (disease-free survival - left, Progression-free interval - middle) and AJCC staging (overall survival - right). De Long p-value estimating difference between two area under curves, significant < 0.05.

**Figure 3.**
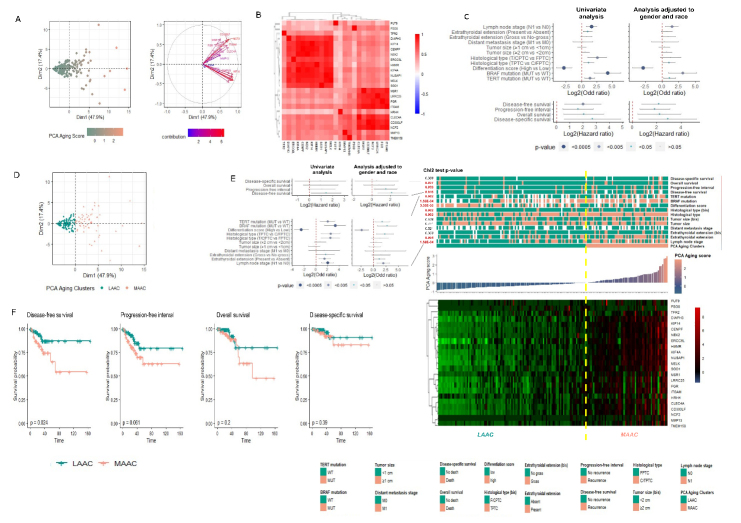
The 23-AC-DEGs stratified aging-tumor samples in two distinct clusters. (A) Principal component analysis representing the significance of the 23 AC-DEGs to explain the heterogeneity observed among the old tumor samples cohort (left) and their correlation (right). (B) heatmap representing their Spearman correlation matrix of the expression of the 23 AC-DEGs in the aging tumor cohort samples. (C) PCA aging score computed from the expression of the 23 DEGs independent of association with tumor features or clinical parameters. (D). Logistic regression analyses between the 23-AC-DEGs PCA Aging score and aggressive clinical parameters, in a univariate and multivariate (adjusted to gender and race) analyses. (E) Stratification of the old tumor cohort in 2 clusters based on the PCA ageing score, LAAC (less aggressive ageing cluster) and MAAC (more aggressive ageing cluster). Chi2 test p-value to evaluate the significant difference of the clinical parameters distribution according to the 2 aging clusters) (right), and by a (left bottom). (F) Kaplan Meier curves representing the survival probability according to the two aging tumor sample clusters for disease-free survival, progression-free interval, overall survival, and disease-specific survival (Log rank test p-value). p-values significant < 0.05.

To identify transcriptome-wide changes in gene expression, DGE analysis was carried out comparing 19 aging tumor samples with their normal counterparts, also adjusting for gender and race (Supplementary Fig. 3A). We performed similar analyses for young (tumor vs normal) samples (Supplementary Fig. 3B) and normal samples (≥55 vs <55) (Supplementary Fig. 3C). Venn diagram analysis was carried out to identify genes dysregulated only in either cohort or common to both (Supplementary Fig. 3D). A total of 197 AC-DEGs and 1 AC-DEmiRNA (miR-9-5p) were found to be unique to the aging-cancer cohort (≥ 55) (Fig. 1A-B, Supplementary Fig. 3D-F). Among the AC-DEGs, we identified potential regulators of the aging transcriptome: three lncRNA (two downregulated [*C6orf176* and *LOC285796*] and one upregulated [*MIAT*]), four pseudogenes (two downregulated [*CETN4P* and *AGAP11*] and two upregulated [*EMR4P* and *SIGLECP3*]), and 15 TFs or co-TFs (one downregulated [*NUPR1*] and 14 upregulated including *FOXM1*, *EZH2*, *E2F8*, *HOXA11*, and *SOX11*). In the aging cohort, 1,656 genes presented a CN gain with a frequency > 2% and had a positive correlation between their expression and their copy number. Of these, 154 genes had a significant positive fold change (log2FC >1) in the aging tumors compared to their normal counterparts. Interestingly, seven of these genes (*ASPM*, *CENPF*, *EXO1*, *KIF14*, *NCF2*, *NEK2*, and *ZNF695)* were characterized as AC-DEGs. They were localized in the q arm of chromosome 1 and presented a copy number gain of 9% in the aging tumors. Copy number loss influenced the expression of 986 genes, and 18 presented a significant negative fold change (log2FC < -1) in aging tumors (Fig. 1A).

Comparing markers of genomic instability between old and young cancer cohorts, aging tumors showed a significantly higher rate of tumor mutational burden/mutation density (Kruskal-Wallis test, p=2 x 10^-16^), number of single nucleotide variant (SNV) neoantigens (p=9.7 x 10^-15^), number of single nucleotide polymorphisms (SNPs) (p=5.2 x 10^-9^), silent (p=6.1 x 10^-12^) and non-silent (p=7.2 x 10^-16^) mutation rate, fraction altered (p=6.7 x 10^-4^), aneuploidy score (p=4.8 x 10^-5^), and homologous recombination defect score (p=2.7 x 10^-3^) (Supplementary Fig. S4).

### Interaction network and functional role of the 198 AC-DEGS/DEmiRNA in aging thyroid tumorigenesis

To identify potential regulatory interactions between AC-DEGs, a protein-protein interaction (PPI) network was built highlighting PPI with Spearman correlation between nodes > |0.5| using the STRING platform. A regulatory network using differentially expressed TFs, lncRNAs, and miRNAs regulating the AC-DEGs was constructed. Only loops between AC-DEGs/TFs/miRNAs, AC-DEGs/TFs/lncRNAs, and DEGs/lncRNAs/miRNAs with Spearman correlation > |0.5| were selected. We observed two subgroups of AC-DEGs interactomes. The first were tightly regulated, through *MELK*, *BRCA2*, *KIF14*, *BUB1*, and *TOP2A*, by five TFs (*NFE2L3*, *RUNX1*, *RUNX2*, *BHLHE40*, and the aging cancer-specific *SOX11*), two LncRNAs (*ST7OT1* and *CDKN2BAS*), and four miRNAs (*miR-21-5p, miR-363-3p, miR-873-5p*, and *miR-138-1-3p*). The second interactome represented by NCF2, CLEC4A, CLEC7A, MSR1, ITGAM, ITGAX, CD300LF, CD68, FGR, and CCR1 were controlled by a plethora of regulating TF/miRNA, TF/LncRNA, and miRNA/LncRNA loops (Fig. 1C).

Next, pathway enrichment/gene ontology analyses were performed to identify specific aging-related cancer roles for the AC-DEGs. We performed a similar analysis in the younger cohort (Fig. 1D, Supplementary Fig. 5A-D). Pathways were selected based on two criteria: First, pathways had to present a significant association with malignancy status, estimated by a logistic regression using the pathway agglomerate z-scores; second, pathways had to be specific, have an opposite behavior, or be significantly different from the other age comparisons. Compared to aging normal tissue, aging tumors were specifically enriched in 20 KEGG pathways, 30 GO-BP (Fig. 1D), 19 GO-MF, and 12 GO-CC (Supplementary Fig. 5A) entries. Based on the correlation between pathways and processes enriched uniquely in the aging cohort, two subclusters of related processes were identified (Fig. 1E). The first subnetwork was related to mitosis progression and DNA damage entries (“mitotic spindle assembly,” p=6.1 x 10^-5^; “regulation of cytokinesis,” p=4.3 x 10^-7^; “Fanconi anemia pathway”, p=7.0 x 10^-8^; “homologous recombination,” p=6.4 x 10^-5^; “cellular response to DNA damage stimulus,” “mitotic cytokinesis,” “mitotic cell cycle”). The second subnetwork was enriched in entries associated with cell cycle regulation and immune response (“Cellular senescence,” p=1.67 x 10^-7^; “natural killer cell mediated cytotoxicity,” p=2.5 x 10^-5^; “negative regulation of inflammatory response to antigenic stimulus,” p=5.1 x 10^-7^; “regulation of cell cycle”). This second subnetwork was also characterized by the Fc-gamma receptor, chemokine, and NF-κB signaling pathways.

### Immune cell infiltration landscape in the cancer-aging samples

The interaction between immune cells and the tumor microenvironment (TME) is important during tumorigenesis and is a key determinant in cancer trajectory and treatment outcomes [50]. To study the immune landscape in aging TC, we estimated the infiltration percentage of 22 immune cells in the TCGA cohort using the CIBERSORT algorithm [33]. Tumors in the aging cohort were enriched in M0 macrophages, mast cells, resting dendritic cells, and regulatory T-cells (Tregs) but also showed a decrease in CD8+ T-cells and T-cell follicular helpers. Young tumors showed an enriched activation of NK cells, dendritic cells, and macrophage M2, and an impoverishment or decrease in B (naïve and memory), activated T-cell CD4 memory, mast cells, and macrophage M1 (Fig. 1F). Taken together, these results highlight specific aging-dependent pathways in thyroid tumorigenesis associated with a deregulation of the cell cycle, cell death, DNA damage repair, and immune response.

### Identification of an aging-cancer and aging-dependent aggressive panel in the aging cohort

Through filtration and internal validation steps in the aging cohort, the 198 AC-DEGsAC-DEmiRNA were filtered to 23 aging-specific and aging-dependent genes associated with aggressive phenotypes (Fig. 2A). A similar workflow applied to the young cohort identified 64 genes as cancer-specific and dependent on age <55 for expression. From univariate and multivariate analysis, the 23 DEGs showed high accuracy in predicting clinical parameters and were significantly associated with a higher chance of the presence of LNM, ETE, undifferentiated state, *BRAF^V600E^* and TERT promoter mutation with coefficient-indexes (C-index) > 0.8 (Fig. 2B-C). Most genes in this panel showed a significant positive fold change associated with higher ATA risk strata, important tumor features and clinical parameters, markers of genomic instability, and immune cell and response indicators (Supplementary Fig. 6A-C). Importantly, significant positive fold change association between genes found in this panel, such as *MSR1*, *FGR*, *LRRC25*, and *CLEC4A* and *BRAF* mutations, were observed. Given the association between the individual 23 AC-DEGs and aggressiveness phenotypes and outcome in aging patients, we tested their potential of prognostication as a unique signature. A risk score, named 23-Risk score, was computed using the expression values of the 23 DEGs weighted by their regression coefficient. We observe that this score could predict with high accuracy the presence of LNM, (OR=5.0, p=7.2 x 10^-8^), ETE (present vs. absent, OR=4.9, p=2.3 x 10^-8^; gross vs. non-gross (OR=3.0, p=7.03 x 10^-6^), distant metastasis (OR=3.8, p=2.0 x 10^-3^), histological type (T/CPTC vs. FPTC, OR=7.09, p=1.1 x 10^-7^; TPTC vs. F/CPTC, (OR=3.0 p=6.47 x 10^-6^), *BRAF^V600E^* (OR=8.6, p=2.4 x 10^-6^) and *TERT* promoter (OR=4.12 p=1.9 x 10^-6^) mutations, and undifferentiated score (OR=5.9, p=1.9 x 10^-7^) with coefficient-indexes (C-index) > 0.8 (Fig. 2B-C).

### A 23 AC-DEGs signature as a prognosis marker for aging thyroid carcinoma patients

Given the association between this 23-gene panel and aggressiveness phenotypes in aging patients, we tested the potential of the derived RISK score in predicting TC stage and reoccurrence risk in comparison to both AJCC and ATA staging and reoccurrence risk markers. First we observe that a significantly higher RISK score was associated with a higher lymph node stage, (p=5.8 x 10 ^-15^), ETE (present vs. absent, p=1.9 x 10^-13^; gross vs. non-gross (p=1 x 10^-5^), distant metastasis stage (p=5.8 x 10^-4^), *BRAF^V600E^* (p=2.7 x 10^-12^) and *TERT* promoter (p=3.5 x 10^-10^) mutations, and undifferentiated score (OR=5.9, p=1.9 x 10^-7^) (Supplementary Fig. 7A-C). Patients with higher RISK scores had significantly lower survival outcome measures than those with lower RISK scores: DFS (HR=2.72 p=1.5 x 10^-12^), PFI (HR=2.7, p=9.5 x 10^-12^), and OS (HR=2.7, p=4.7 x 10^-7^) (Fig. 2D). We observe that the 23-AC-DEGs signature showed significantly superior performance in predicting patient outcomes compared to the ATA approach for recurrence prediction. The C-index estimates were 0.95, 0.86, and 0.95 for DFS, PFI, and OS, respectively. A DeLong test comparing the AUCs for both risk prediction methods validated the significance of the more accurate predictions of the 23 DEG signature with all p-values < 0.05 (Fig. 2E). These results strongly suggest that the 23 AC-DEG panel is a robust prognostic marker panel that can complement established clinical guidelines and help to improve staging and risk evaluation.

### The 23 AC-DEGs stratified the aging tumors into two clusters: a more aggressive aging cluster (MAAC) and a less aggressive aging cluster (LAAC)

A PCA aging score was calculated summarizing the weight of the contribution of each AC-DEG to explain the samples heterogeneity. Compared to the RISK scores computed previously, the PCA aging score was directly calculated from the expression of the 23 DEGs, independent of their association with clinical parameters. Unsupervised clustering of the 23 AC-DEGs stratified the aging tumors into two subclusters with varying PCA scores. These genes could explain more than 65% of the heterogeneity observed across the aging tumors. Furthermore, the expression of these genes was positively correlated (Fig. 3A-B). Each subcluster had a distinct association with clinically aggressive phenotypes, immune cell infiltration, and genomic profiles (Supplementary Fig. 6A-C). The KIF4A subgroup, including *KIF4A*, *KIF14*, *MELK*, *NUSAP1*, *SGOL1*, *HMMR*, *CENPF*, *ERCC6L*, *DIAPH3*, *NEK2*, and *TRF2* (sorted according to their contribution to sample heterogeneity), showed the strongest association with aggressiveness markers (Supplementary Fig. 6A) and immune cell infiltration (Supplementary Fig. 6C). Conversely, the similarly sorted *NCF2* subgroup, including *NCF2*, *CLEC4A*, *ITGAM*, *LRRC25*, *CD300LF*, *FGR*, *TMEM158*, *MMP13*, *MSR1*, and *HRH4*, displayed a significant independent association with markers of genomic instability (Supplementary Fig. 6B). We observed that the PCA aging score was also associated with aggressiveness markers in univariate and multivariate (adjusted for gender and race) regression analyses (Fig. 3C). A significantly higher PCA aging score was observed in patients with LNM (p=2.0 x 10^-3^), tall-cell aggressive histological variant (p=2.6 x 10^-2^), undifferentiated state (p=2.1 x 10^-4^), *BRAF^V600E^* (p=5.0 x 10^-3^), and *TERT* promoter mutations (p=1.7 x 10^-2^), DFS (p=6.0 x 10^-3^), PFI (p=3.0 x 10^-3^), OS (p=8.0 x 10^-3^), and DSS (p=1.6 x 10^-2^). Additionally, a higher PCA aging score corresponded with poorer survival outcomes (Supplementary Fig. 8A, B). Based on this score, samples were stratified into two clusters: a MAAC with a PCA aging score > 0 and a LAAC with a PCA aging score < 0, 0 being the mean PCA aging score (Fig. 3D). These two clusters significantly differentiated samples according to their clinical aggressive phenotype based on regression analysis and Pearson’s Chi-squared test (Fig. 3E). Furthermore, MAAC tumors presented a higher risk of recurrence with a five-year survival rate of 65% (CI-95%=40-82% vs. 87% [CI-95%=75-94%], p=2.4 x 10^-2^) (Fig. 3F). A comparable analysis for the 64 DEGs identified as unique to the young cohort was carried out and showed a significant association between the expression of these genes and clinical aggressiveness-related phenotypes in both univariate and multivariate analyses (Supplementary Fig. 9A-D). We also observe that PCA scores computed from the DEGs-YCs stratified young samples into two clusters: a less aggressive young cluster (LAYC) and a more aggressive young cluster (MAYC). However, there were no differences in survival outcomes between the two clusters (Supplementary Fig. 9E). These results suggested that two aging subpopulations with distinct tumor pathology and patient outcome can be identified.

**Figure 4.**
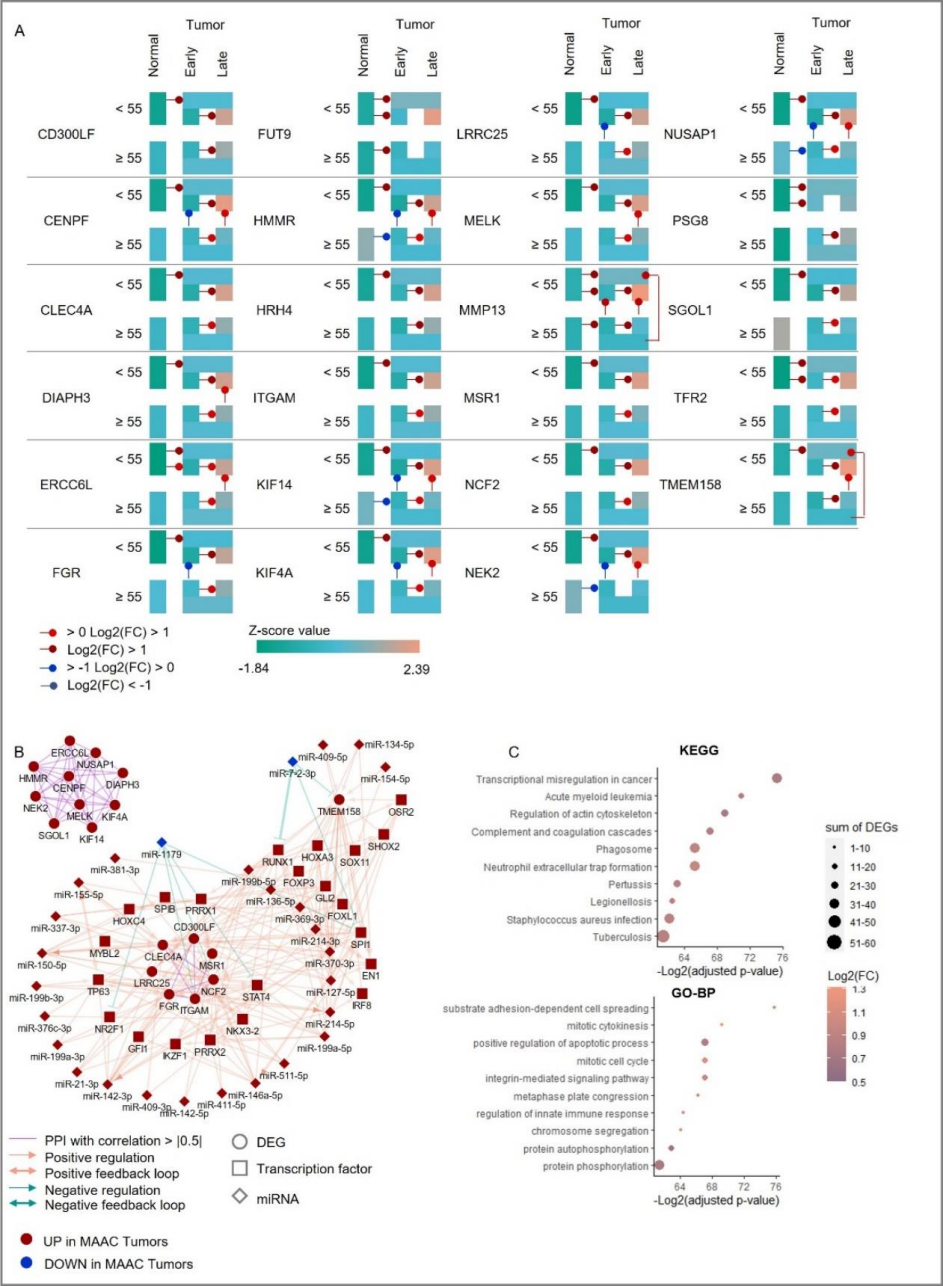
Transcriptomic landscape induced by the 23 AC-DEGs. (A) Heatmap representing the z-score mean of 23 AC-DEGs expression in each cluster. Arrows represent a significant FC. (Boxplot visualization of the expression of the 23-AC-DEGs across the whole thyroid samples is shown in Supplementary Figure 17A). (B) PPI and regulatory network of TFs and miRNAs strongly regulating the 23 DEGs. Only TF/miRNA/DEG loops with spearman correlation > |0.5| were selected (no LncRNA loops identified). (C) Dot plots representing the 10^th^ first most significant KEGG (top) and GO-BP (bottom) pathways selected after an enrichment analysis comparing MAAC vs LAAC, according to their fold change adjusted p-value. Only pathways unique of tumor-aging or significantly different compared to the younger comparison (MAYC vs LAYC) and including the 23 DEGs-AC were finally selected.

### Expression and functional role of the 23 AC-DEGs in aging tumor progression to aggressiveness

To decipher the role of the 23 AC-DEGs in aging tumor progression, we analyzed the expression of the 23 AC-DEGs in the normal thyroid and in early and late tumor stages as well as in the identified aggressiveness clusters (Fig. 4A, Supplementary Fig. 10A). All but two of these genes (*FUT9* and *PSG8)* presented a significant upregulation in the MAAC samples compared to the LAAC (Supplementary Fig. 10A-B). Only five of the 23 AC-DEGs showed a FC > |1| when MAYCs were compared with LAYCs, confirming that the expression of genes in this panel and their potential role appear to be more crucial to aggressiveness/progression in aging cohorts than younger cohorts (Supplementary Fig. 10C). Protein-protein network analysis identified two distinct clusters from the 23 DEGs with correlated interactions, confirming the distinction between the two subgroups of genes previously identified (Fig. 4B). The *NCF2* subgroup formed a denser network involving 27 miRNAs and 23 TFs, creating 98 regulatory loops. Next, pathway enrichment analyses were performed between MAAC and LAAC samples, and only entries with the 23 DEGs were retained (Fig. 4C, Supplementary Fig. 10D). As previously described, similar pathways observed in the young comparison were removed. We observed a strong enrichment of entries related to cell cycle and mitosis progression, including “mitotic cytokinesis” (p=1.49 x 10^-21^), “mitotic chromosome condensation” (p=3.64 x 10^-19^), “mitotic sister chromatid segregation” (p=6.9 x 10^-19^), “chromosome segregation” (p=5.1 x 10^-20^) and “mitotic cell cycle” (p=6.4 x 10^-21^) etc. (Fig. 4C).

### Aging-related transcriptomic landscape in normal thyroid tissue

**Figure 5.**
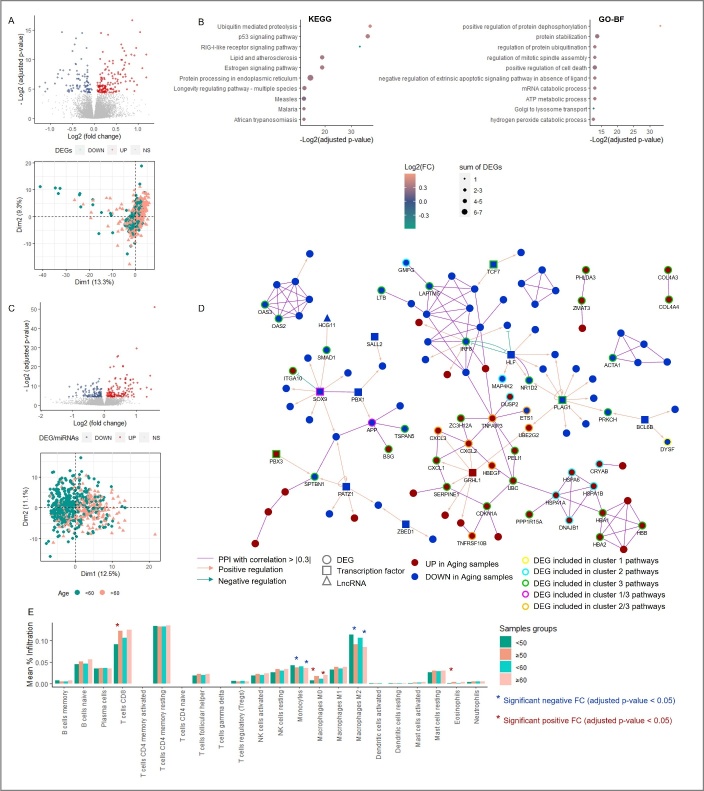
Ageing related transcriptomic landscape in thyroid normal tissue. (A) Volcano (left) and corresponding PCA plots of genes (right) after Differential Expressed Genes analyses in the aging normal cohort (≥50 vs <50, 445 old vs 208 samples - 103 and 118 down and up-regulated genes). (B) Dot plots representing the adjusted p-value of KEGG pathways (left) and GO-BP entries (right) differently enriched in the old (≥50) cohort. The 10 first most significant are described. (C) Volcano (left) and corresponding Principal Component Analysis (PCA) plots of genes (right) after Differential Expressed Genes (DEGs) analyses in the aging normal cohort (≥60 vs <60, 234 old vs 445 samples- 89 and 143 down and up-regulated genes. (D) PPI and regulatory network of the aging DEGs. Only edges with a spearman correlation > |0.3| were selected. (E) Bar plot representing CIBERSORT immune cell infiltration mean in the young and old normal samples. * adjusted p-value < 0.05. FC, fold change.

To decipher the impact of aging on normal thyroid physiology, all 653 normal thyroid samples from the GTEx dataset tissue samples were analyzed and adjusted to gender and Hardy score. Since age in the GTEx dataset was categorized by decade and did not match the <55 and ≥ 55 distinctions in the AJCC guidelines, we first compared gene expression between samples older and younger than 50 (445 vs. 208 samples). A total of 223 DEGs (109 downregulated and 114 upregulated) were identified in the aging cohort (Fig. 5A). Interestingly, from PCA visualizing the sample variance explained by the DEGs, the first two PCs explained only 22.6% of the heterogeneity observed among the normal samples for both comparisons (Fig. 5A). The 223 DEGs were used as input for KEGG and gene ontology (GO) enrichment pathway analyses. (Fig. 5B-D, Supplementary Fig. 11A). Further selection was performed after logistic regression computing the pathways’ agglomerate z-score to identify the significant aging-associated pathways. The most enriched KEGG pathways were related to protein maturation, trafficking, and degradation, including “protein processing in endoplasmic reticulum” (OR=9.3, p=4.6 x10^-8^) and “endocytosis” (OR=1.8, p=1.3 x 10^-2^), “longevity regulating pathway” (OR=6.5, p=2.8 x 10^-6^), “antigen processing and presentation” (OR=4.2, p=7.6 x 10^-5^), and “estrogen signaling pathway” (OR=5.8, p=4.6 x 10^-8^). The enriched GO-BP (biological processes) were related to protein maturation with “protein refolding” (OR=4.2, p=5.35 x 10^-5^), “response to unfold protein” (OR=9.5, p=4.4 x 10^-5^), “regulation of protein ubiquitination” (OR=3.2, p=4.4 x 10^-5^) entries (Fig. 5B). This suggests a link between aging and unfolded protein response, protein maturation and stability. A similar analysis was performed with a different age comparison (≥ 60 vs. < 60) to decipher the transcriptomic landscape around age 55 (Fig. 5C, Supplementary Fig. 11B-D). A PPI network (based on Spearman correlation > |0.3| between nodes) was created between the DEGs and differentially expressed TFs including IRF8, HLF, SOX9, GRHL1, BCL6B, and PATZ1. Two interesting PPI subclusters could be highlighted, the chemokines (with CXCL1, CXCL2, and CXCL3) and the heat shock proteins (with HSPA1A, HSPA1B, HSPA6 and DNAJB1) (Fig. 5D).

Immune infiltration in the microenvironment is a dynamic, and aging is a known modulator of this process [51, 52]. Analysis of microenvironmental heterogeneity in the GTEx data revealed that older tissues were associated with an increase in infiltration with M0 macrophages (p=5.0 x 10^-3^), CD8+ T-cells (p=1.2 x 10^-5^), and eosinophils (p=2.5 x 10^-2^), along with a decrease in M2 macrophages (p=1.2 x10^-7^) and monocytes (p=5.0 x 10^-3^) (Fig. 5E). Taken together, these results strongly suggest that aging itself does not induce a deep transcriptomic change in the thyroid, as shown by the small number of DEGs. Second, aging seems to primarily alter protein stability [53] and the endosome machinery [54], suggesting that endoplasmic reticulum stress occurs during the thyroid aging process, leading to the deregulation of several survival and proliferative pathways [55]. The aging thyroid gland seems to present a specific immune cell infiltration landscape that may protect against cancer given its enrichment with M0 macrophages and CD8+ T-cells [56].

**Table 1:** Table summarizing distribution of the clinical parameters across the 4 clusters identified.

							LAYC	MAYC	LAAC	MAAC
						Number of samples	189	132	103	57
						Percentage	39.3	27.4	21.4	11.9
		Number of samples					Percentage of samples			
		LAYC	MAYC	LAAC	MAAC	Chi^2^ test p-value	LAYC	MAYC	LAAC	MAAC
Lymph node metastasis	N0	90	41	67	21	1.71E-07	41.1	18.7	30.6	9.6
	N1	77	80	26	33		35.6	37.0	12.0	15.3
Tumor size	< 1 cm	62	36	32	20	0.664	41.3	24.0	21.3	13.3
	≥ 1 cm	127	96	71	37		38.4	29.0	21.5	11.2
	< 2 cm	137	103	75	35	0.135	39.1	29.4	21.4	10.0
	≥ 2 cm	52	29	28	22		39.7	22.1	21.4	16.8
Extrathyroidal extension	Absent	151	80	66	25	1.29E-07	46.9	24.8	20.5	7.8
	Present	33	50	30	32		22.8	34.5	20.7	22.1
	No-Gross	184	128	90	47	1.31E-08	41.0	28.5	20.0	10.5
	Gross	0	2	6	10		0.0	11.1	33.3	55.6
Distant metastasis	M0	97	81	57	31	0.219	36.5	30.5	21.4	11.7
	M1	3	1	2	3		33.3	11.1	22.2	33.3
Histological type	FPTC	62	4	30	4	1.99E-11	62.0	4.0	30.0	4.0
	C/TPTC	123	124	72	53		33.1	33.3	19.4	14.2
	C/FPTC	183	113	97	44	1.30E-07	41.9	25.9	22.2	10.1
	TPTC	2	15	5	13		5.7	42.9	14.3	37.1
BRAF mutation	WT	56	3	28	3	2.07E-14	62.2	3.3	31.1	3.3
	MUT	64	93	37	39		27.5	39.9	15.9	16.7
TERT mutation	WT	145	98	67	27	9.95E-14	43.0	29.1	19.9	8.0
	MUT	2	5	11	18		5.6	13.9	30.6	50.0
Differentiation score	Low	54	93	28	34	9.26E-18	25.8	44.5	13.4	16.3
	High	98	17	54	12		54.1	9.4	29.8	6.6
Disease-free survival	Non-recurrence	177	120	89	37	0.001	41.8	28.4	21.0	8.7
	Recurrence	11	13	8	12		25.0	29.5	18.2	27.3
Overall survival	Alive	188	133	97	47	1.39E-10	40.4	28.6	20.9	10.1
	Dead	0	0	6	10		0.0	0.0	37.5	62.5
Progression-free interval	Non-recurrence	177	120	91	43	7.88E-04	41.1	27.8	21.1	10.0
	Recurrence	11	13	11	14		22.4	26.5	22.4	28.6
Disease-specific survival	Alive	188	133	98	48	1.57E-04	40.3	28.5	21.0	10.3
	Dead	0	0	3	4		0.0	0.0	42.9	57.1
number of non-aggressive enrichments							11	4	4	0
number of aggressive enrichments							0	7	5	12
Ratio (negative: non-aggressive parameter enrichment; positive: aggressive parameter enrichment)							-0.92	0.25	0.08	1.00

### Aging-related aggressive thyroid hierarchy

**Figure 6.**
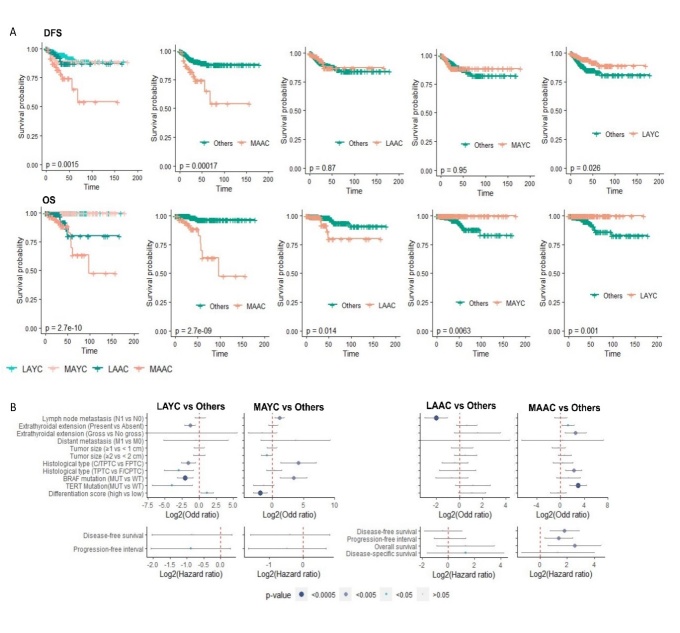
The four thyroid cancer clusters aggressive hierarchy. (A) Kaplan-Meier curves plotting survival probability according to DFS and OS for the four samples clusters (left), and each cluster compared to the 3 others (right) (log rank test p-value). (B) Plots showing multivariate logistic regression analyses (adjusted to gender, race, lymph node metastasis, tumor size, extrathyroidal extension, and histological type) testing, from left to right, LAYC vs Other, MAYC vs Others, LAAC vs Others, and MAAC vs Others. OS and DSS Cox regression analyses were not shown for LAYC and MAYC as not death event was present in these clusters. CPTC, Classical variant papillary thyroid carcinoma (PTC); DFS, Disease-free survival; Disease-specific survival; FPTC, Follicular variant PTC; MUT, Mutant; OS, Overall survival; TPTC, Tall-cell variant PTC; WT, Wild-type. p-value significant < 0.05.

To better understand the degree to which aging modulates TC aggressiveness, we compared the identified (aggressiveness) clusters and ranked cluster-based enrichment with aggressiveness phenotypes. Table 1 shows the distribution of clinical parameters in the four clusters. Pearson’s chi-squared test was used to estimate enrichment with non-aggressive (blue) and aggressive (red) clinical parameters. All parameters except tumor size and distant metastasis presented significantly different enrichment between the four clusters. After calculating a ratio of enrichment between the aggressive and non-aggressive parameters, we determined that MAAC was the most aggressive, followed by MAYC and LAAC, with LAYC being the least aggressive. Univariate and multivariate analyses confirmed this aggressive hierarchy. Finally, we observed that only MAAC significantly presented tumors with a higher risk of recurrence. Indeed, MAAC had a three- to six fold higher risk of a worse outcome than MAYC (DFS, HR=3.1, p=5.0 x 10^-3^; PFI, HR=2.6, p=5.0 x 10^-3^), LAYC (DFS, HR=5.7, p=4.9 x 10^-4^; PFI, HR=5.1, p=5.0 x 10^-4^), and LAAC (DFS, HR=3.8, p=1.4 x 10^-2^) (Fig. 6A-B, Supplementary Fig. 12). The most aggressive cluster, MAAC, was specifically enriched in samples with ETE (gross vs. non-gross, OR=6.0, p=4 x 10^-2^; present vs. absent, OR=2.5, p=1.3 x 10^-2^), presence of tall-cell variant histological subtype (OR=4.7, p=1.0 x 10^-3^), and *TERT* promoter mutation (OR=7.9, p=3.0 x 10^-5^) (Table 1, Fig. 6A, B). The genomic landscape difference between MAAC and LAAC was further investigated. The MAAC samples were significantly enriched in copy number gain in 26 loci on chromosome 1, with a frequency greater than 20% (Supplementary Fig. 13A). A total of 41 genes holding a significant copy number gain were significantly upregulated in the MAAC compared to the LAAC samples, and three AC-DEGs (*ASPM*, *CENPF*, *NCF2)* had a copy number gain of 21%. Globally, genomic alterations were found across chromosomes, except for 13p, 14p, 18p, and 22p loci. Loci in 1p/1q were found altered in all clusters except LAYC and were of significantly higher frequency in MAAC, suggesting that 1p/1q loci are related to tumor aggressiveness as previously noted (Fig. 6B, Supplementary Fig. 13A-B). Pathway enrichment was estimated from the selected LAAC-specific DEGs, and two clusters of pathways were identified (Fig. 13B). The first subnetwork was enriched in entries related to cell death (“positive regulation of intrinsic apoptotic signaling pathway,” p=2.0 x 10^-3^; “Apoptosis - multiple species,” p=1.0 x 10^-3^) etc., and impoverished in entries related to immune response (“platelet activation,” p=1.9 x10^-8^; “T cell co-stimulation,” p=1.0 x 10^-2^ etc.). The second subnetwork showed an enrichment of entries related to the metabolism and longevity (“positive regulation of glucose import,” p=3.4 x 10^-11^; “positive regulation of glycogen biosynthetic process,” p=3 x 10^-3^; and “longevity regulating pathway,” p=2.4 x 10^-7^ etc. Among the 2357 DEGs, 218 genes were integrated in a high correlated interactome and 643 were tightly controlled by a 41 miRNA/19 LncRNA/59 TF regulatory network (Supplementary Fig. 13C-E).

**Figure 7.**
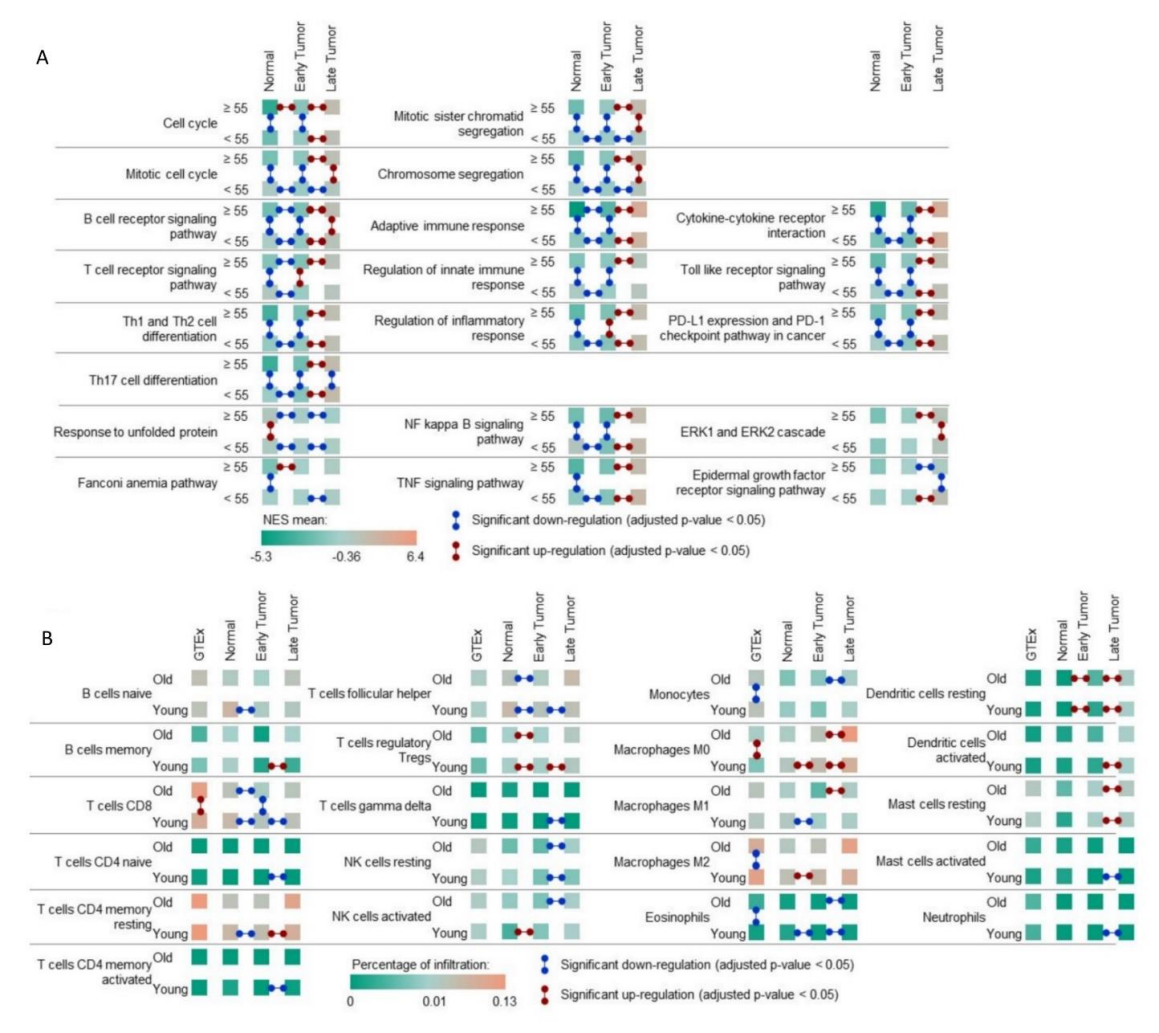
The global aging landscapes. (A-B) Heatmap representing the mean of significant pathway NES score (A) and immune cell infiltration (B) in normal tissue and each tumor stage. Arrows represent a significant FC (red, positive; blue, negative).

### Pathway enrichment across the six thyroid clusters

In previous sections, we evaluated the signaling pathway hallmarks and the immune cell infiltration landscape in normal and cancerous thyroids in young and old cohorts independently (Fig. 1C-F). Because each comparison was performed independently, we were not able to run pathway enrichment analyses in all samples, thus hindering our understanding of the molecular processes involved in the transition from normal thyroid to tumor induction and tumor progression in both young and old patients. To respond to this limit, enrichment analysis of all pathways selected in the different comparisons and all samples were run and statistical analyses were performed. Fig. 7A and Supplementary Fig. 14 show the pathway entries presenting a concordance between the two score analyses. We observe a significant upregulation and positive enrichment of cell cycle pathways (or related processes) in the transition from normal tissue to late tumor stage in patients ≥ 55; however, upregulation and increased positive enrichment in these processes could only be seen when early-stage tumors progress to late-stage in samples aged < 55. Mitotic cell cycle and chromosome segregation were only significantly upregulated and positively enriched in early to late tumors in ≥ 55-year-olds but not in < 55-year-old patients. Interestingly, we observed that immune response-related processes such as B and T cell receptor signaling, helper T cell differentiation, adaptive immune response, and regulation of inflammatory response pathways were downregulated and negatively enriched when normal tissues transitioned to early tumors in both age categories. The cell cycle and mitosis progression entries were also confirmed as hallmarks of aging late tumors, associated with ERK1/2 cascade and EGFR signaling was upregulated in the young late tumors and downregulated in the aging late tumors (Fig. 7A). This strongly suggests that key processes involved in immune surveillance for tumors are not active or activated in early thyroid tumorigenesis. We also observed a downregulation in infiltration with CD8+ T cells and activated natural killer and follicular helper T cells in the thyroid of aged patients in the transition from normal to early tumors (Fig. 7B). Interestingly, the induction of the unfolded protein response was characteristic of the aging normal samples. Indeed, the entry was overexpressed in the aging normal samples and decreased during tumor progression.

## DISCUSSION

In this study, we leveraged the availability of multi-omics datasets and robust statistical methods to uncover molecular markers of age-related discrepancies in the clinical presentation of TC. Our study highlights how the confluence of the hallmarks of aging and TC may drive the acquisition of phenotypes and the transition from indolent to more aggressive TC and consequently poorer survival outcomes. With aging, we observed a significant shift towards metastasis, ETE, large tumor size, TERT mutation, increased mortality, and other indicators of poor prognosis, most evident at age ≥ 55. This suggests that 55 is the age at which the interplay of malignancy drivers and cancer phenotypes is at a steady state but leans towards more aggressiveness. Furthermore, we identified a higher rate of single nucleotide polymorphisms in tumors from aging patients compared to younger patients. Defects in DNA replication and repair, spontaneous deamination of methyl-cytosine to thymine, and longer life history of exposure to cell-intrinsic and external mutagens could explain this observation [57]. While we observe that CNVs are present on multiple chromosomes with gains or losses, aging-dependent aggressiveness was only strongly associated with changes in chromosome 1p/q. Our study, like Chatsirisupachai et al. [58], did show a significant positive association between aging and the occurrence of somatic copy number alteration in TCs. This includes gains on chromosome arms 1p, 5p and losses on arms 9q and 13q identified in that study and ours. Even more, our study identified specific gains on the 4p, 6p, 14q, 19p/q, and 20p/q loci and specific loss on the 2p/q, 8p/q, 10p/q, 11p/q, 16q, 17p, 19p, and 21p/q loci that could play a role in aging-dependent tumorigenesis. Previous studies have linked gains/losses on chromosome 1p/q and 22 to more aggressive PTC, but aging was not identified as a mediator of this observation perhaps due to a utilization of age 45 as a cut-off aggressiveness analysis in that study [59]. Important losses in chromosomal arms with tumor suppressor genes such as *PTEN*, *CDKN2A*, and *TP53* were identified in the aging cohort, potentially explaining the increase in incidence of this cancer type in aging individuals. Increasing evidence suggests that a higher burden of copy number alterations is prognostic of reoccurrence and mortality and can partially explain higher death rates in older patients [60]. Copy Number changes were linked to the expression of aging-specific and aggressiveness-associated genes such as the transcription factor ZNF695; the serine-threonine kinase NEK2; cell-division regulators such as CENPF, EXO1, and ASPM; and extracellular matrix proteins such as MMP19. CENPF is already known as thyroid carcinoma biomarker, involved in migration and proliferation[61]. Interestingly, anaplastic TC, the most aggressive histological TC is enriched in genes involved in the mitosis progression including *CENPF*, *NEK2* and *NUSAP1 [62, 63]*. We also identified several aging-dependent TFs that are upregulated in TCs, such as FOXM1 and SOX11, both of which have fundamental roles in tumorigenesis and aggressiveness. FOXM1 is a spatiotemporally expressed master TF activated by aberrant KRAS signaling to drive cancer initiation, self-renewal, and proliferation. Elevated expression of this TF is associated with poor prognosis in solid tumors, and its silencing has been demonstrated to decrease the invasiveness of TC cells [64, 65]. SOX11 is also a key regulator of organogenesis and embryogenesis, with elevated expression in mesenchymal and neural progenitor cells. Elevated expression of this TF has been observed in an array of solid tumors [66], and its miR-211-5p-mediated downregulation is associated with decreased proliferation and migration in TC cells.

Our analysis also reveals an aging-dependent remodeling of the TME at both onset and late stages. In aging samples, we confirm a previously reported decrease in infiltration with immune surveillant CD8+ T cells [67] and show that significantly decreased activation of anti-neoplastic immune response pathways may drive age-related transition to aggressiveness. Questions remain regarding the prognostic utility of immune cell infiltration in most cancer types, but we provide evidence that immune interactions are important to thyroid tumor trajectory and the treatment response [67, 68]. Interestingly, we did not observe a significant association between increase in *BRAF* mutation (a prognosis marker) and aging. Evidence in the literature on this differs between studies; some report an increase in *BRAF^V600E^* with age [69, 70] and others [71, 72] show no association. This confusion suggests the need for more precise prognostic markers that factor in aging to improve prognosis. A rigorous approach was used in this study to identify prognostic panels of aging-specific and aging-dependent aggressiveness marker genes in adult and young cohorts, respectively. We also show that scores based on these genes demonstrated better performance than both AJCC staging and ATA risk stratification criteria in predicting aggressiveness and determining reoccurrence risk. Internal and external validations were further performed to test the accuracy of the 23-AC-DEGs panel for TC prognostication (Supplementary Fig. 15-20).

Furthermore, high-level classification of patients into low and high aggressiveness clusters can be accomplished using these panels. Thus, our results have the potential to significantly improve prognosis and outcome prediction in clinical use.

Unlike previous studies [3, 4, 58] which have adopted a broad analysis approach to understanding the influence of aging on cancer, our study focuses on TC, for which aging is a prognostically relevant criterion. We comprehensively highlight the molecular pathways and signatures behind the influence of aging on the development of aggressiveness-related phenotypes. A global, pan-cancer evaluation of the molecular drivers of cancer, while useful, often misses cancer-type-specific features due to methodological issues, differences in platforms for data generation, non-uniformity in the histopathological characterization, incomparability of tumor grades, and measurement of clinical outcomes such as treatment response and survival [73].

Overall, we show that aging compounds the effects of genomic instability, transcriptional alterations, cellular interactions with tumors, and important signaling pathways in TC, thereby accelerating metastasis and aggressiveness. Our results do not suggest that aging alone can drive TC onset and aggressiveness; rather, clinical presentation and outcomes of cancers are often dependent on a wide array of tumor-host factors ranging from demographic characteristics to factors such as the cancer origin/cell type, genetic history, mutation profile/burden etc. However, our findings have wide-reaching implications for improving how aging as a continuous biological process is viewed as a powerful modifier in cancer studies. It also creates opportunities for more personalized clinical care based on a more thorough knowledge of the tumor cellular landscape.

## Supplementary Materials

The Supplementary data can be found online at: www.aginganddisease.org/EN/10.14336/AD.2022.1021.
