# Supplementary material for: An Integrative Multi-Omics Analysis of The Molecular Links between Aging and Aggressiveness in Thyroid Cancers
Source: Aging Dis. 2023 Jun 1;14(3):992–1012. doi: 10.14336/AD.2022.1021 (PMC10187705; doi:10.14336/AD.2022.1021)
Supplement: Supplementary file 1 — The Supplementary data can be found online at: www.aginganddisease.org/EN/10.14336/AD.2022.1021. [file AD-14-3-992-s.pdf]

SUPPLEMENTARY DATA

An Integrative Multi-Omics Analysis of The Molecular Links between Aging and Aggressiveness in Thyroid Cancers

Emmanuelle Ruiz[1#], Emad Kandil[2#*], Solomon Alhassan[2], Eman Toraih[2], Youssef Errami[2], Zakaria Y. Abd Elmageed[2,3], Mourad Zerfaoui[2*]

© 2022. Ruiz E et al. Published online at http://www.aginganddisease.org/EN/10.14336/AD.2022.1021

SUPPLEMENTARY DATA

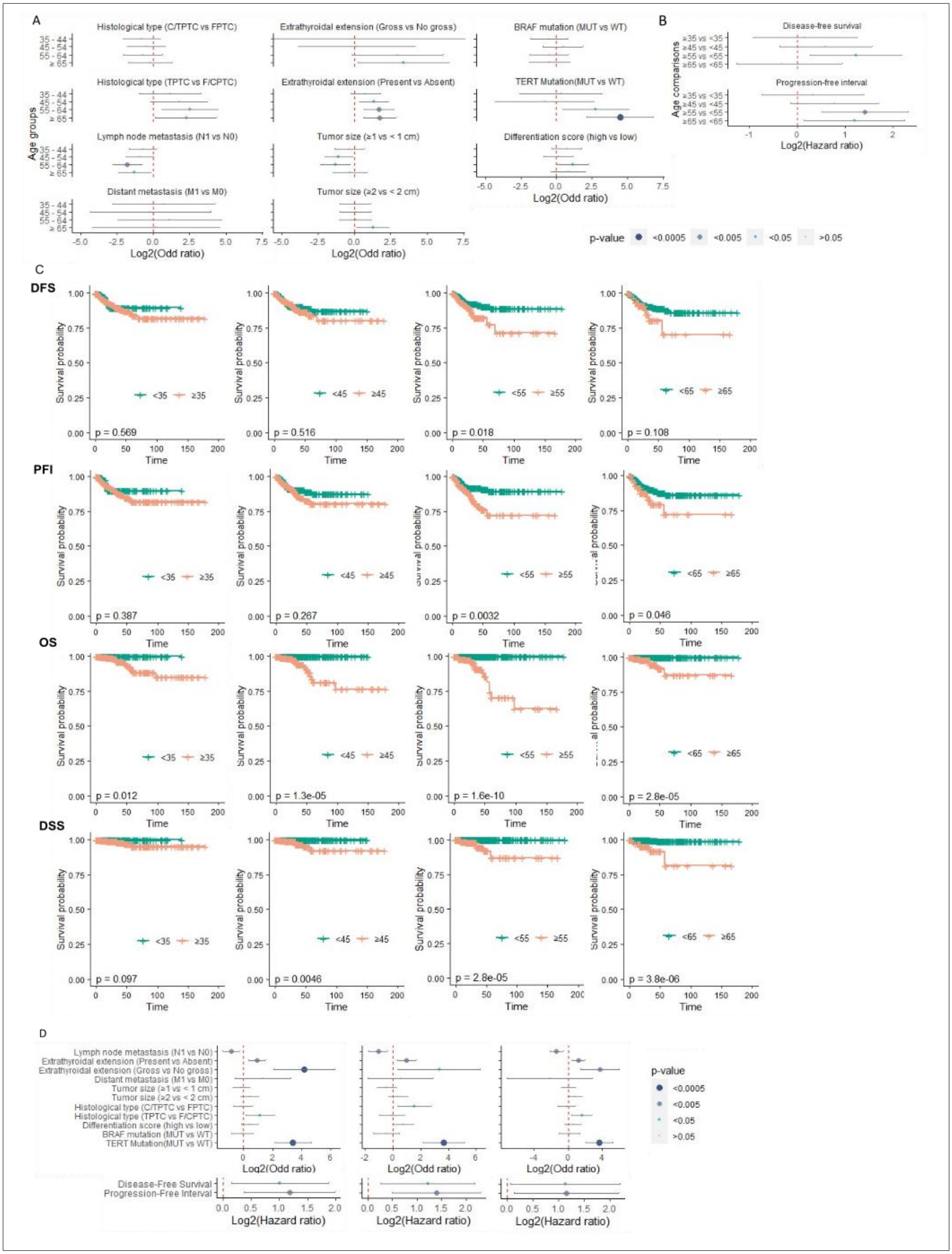

© 2022. Ruiz E et al. Published online at http://www.aginganddisease.org/EN/10.14336/AD.2022.1021

SUPPLEMENTARY DATA

Supplementary Figure 1. Age is a prognosis marker in thyroid carcinoma. A-B. Plots showing gender and race adjusted multivariate logistic regressions performed for the age groups according to clinical parameters (**A**) and Cox regressions for age comparisons according to DFS and PFI (**B**) Cox regression: likelihood ratio test p-value. OS and DSS analyses were not presented as samples with age younger than 55 did not present death event. **C**. Kaplan-Meier curves plotting survival probability for 4 ages comparisons according to DFS, PFI, OS, and DSS (log rank test p-value). **D**. Plots representing logistic regressions performed for the age comparison "≥55 vs <55" according to clinical parameters, as univariate (left), adjusted to gender and race (middle), and adjusted to gender, race, lymph node metastasis, extrathyroidal extension, tumor size, and histological type (right) models. Significant values of the last multivariate analysis are shared in the result section. CPTC, Classical variant papillary thyroid carcinoma (PTC); DFS, Disease-free survival; Disease-specific survival; FPTC, Follicular variant PTC; MUT, Mutant; OS, Overall survival; PFI, Progression-free interval; TPTC, Tall-cell variant PTC; WT, Wild-type. p-value significant < 0.05

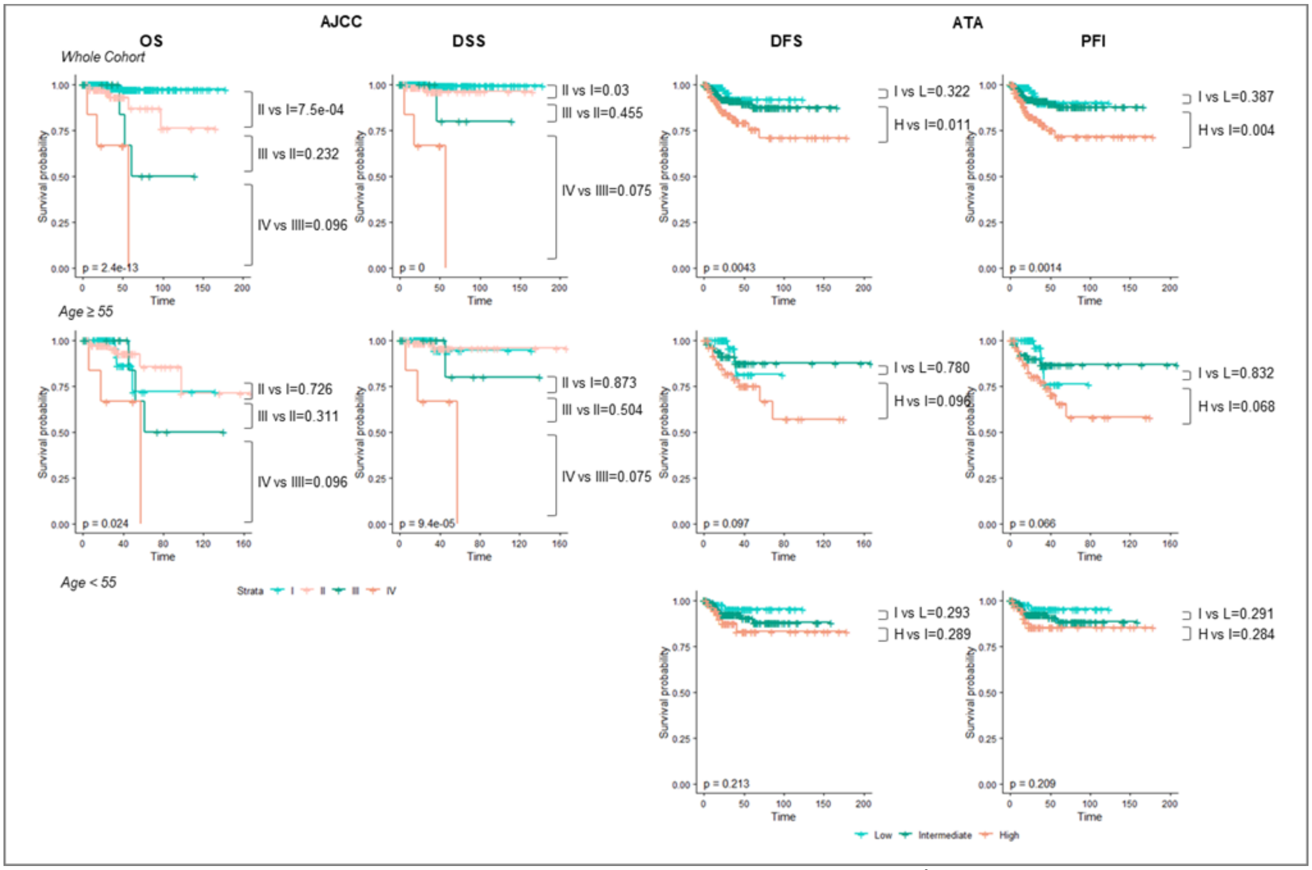

Supplementary Figure 2. Kaplan-Meier curves plotting the survival probability of the AJCC 8th edition staging (left) for OS and DSS, and the ATA risk stratification (right) for DFS and PFI, with the whole cohort (top), old cohort (middle), and the young cohort (bottom). OS and DSS Kaplan Meier curves not shown for the young cohort as there is not samples presenting death event. Log-rank test p-value significant if < 0.05. DFS, Disease-free survival; DSS, Disease-specific survival; H, High risk; I, Intermediate risk; L, Low risk; OS, Overall survival; PFI, Progression-free interval.

© 2022. Ruiz E et al. Published online at http://www.aginganddisease.org/EN/10.14336/AD.2022.1021

SUPPLEMENTARY DATA

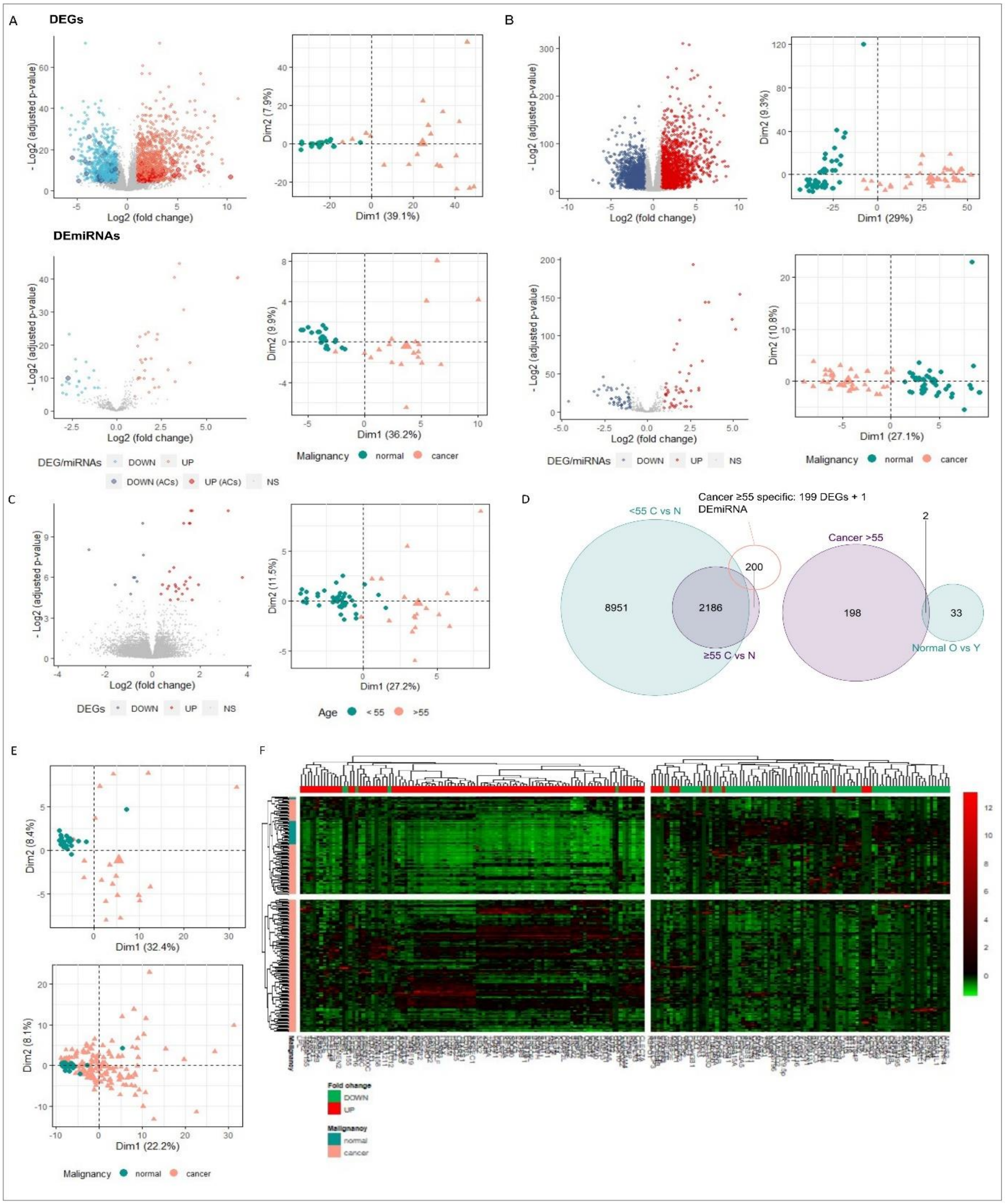

Supplementary Figure 3. A network 197 DEGs and 1 DEmiRNA is specially deregulated in ageing thyroid cancer. A-C. Volcano and corresponding Principal Component Analysis (PCA) plots of genes (left-top) and miRNAs (left-bottom) after Differential Expressed Genes (DEGs) analyses in the aging (**A**), younger (**B**), and normal cohort (**C**) (Old:19 cancer vs 19 normal counterpart – 994 and 1343 down and up-regulated genes and 20 and 29 down and up-regulated miRNAs in cancer; Young: 38 cancer vs 38 normal counterpart – 1794 and 1775 down and up-regulated genes and 46 and 45 down and up-regulated miRNAs in cancer; Normal: 19 old vs 38 young – 8 and 26 down and up-regulated in aging samples). No significant miRNAs were identified in the normal cohort analysis. **D.** Venn diagram summarizing the extraction of specific aging-cancer DEGs/DEmiRNAs **E.** PCA plotted for the DEGs-AC and DEmiRNA-AC in the aging counterpart (top) and whole (bottom) cohort. **F.** Heatmap representing a hierarchical clustering of the whole aging cohort samples according to the 197 DEGs-AC and 1 DEmiRNA-AC. AC, Aging-cancer specific.

© 2022. Ruiz E et al. Published online at http://www.aginganddisease.org/EN/10.14336/AD.2022.1021

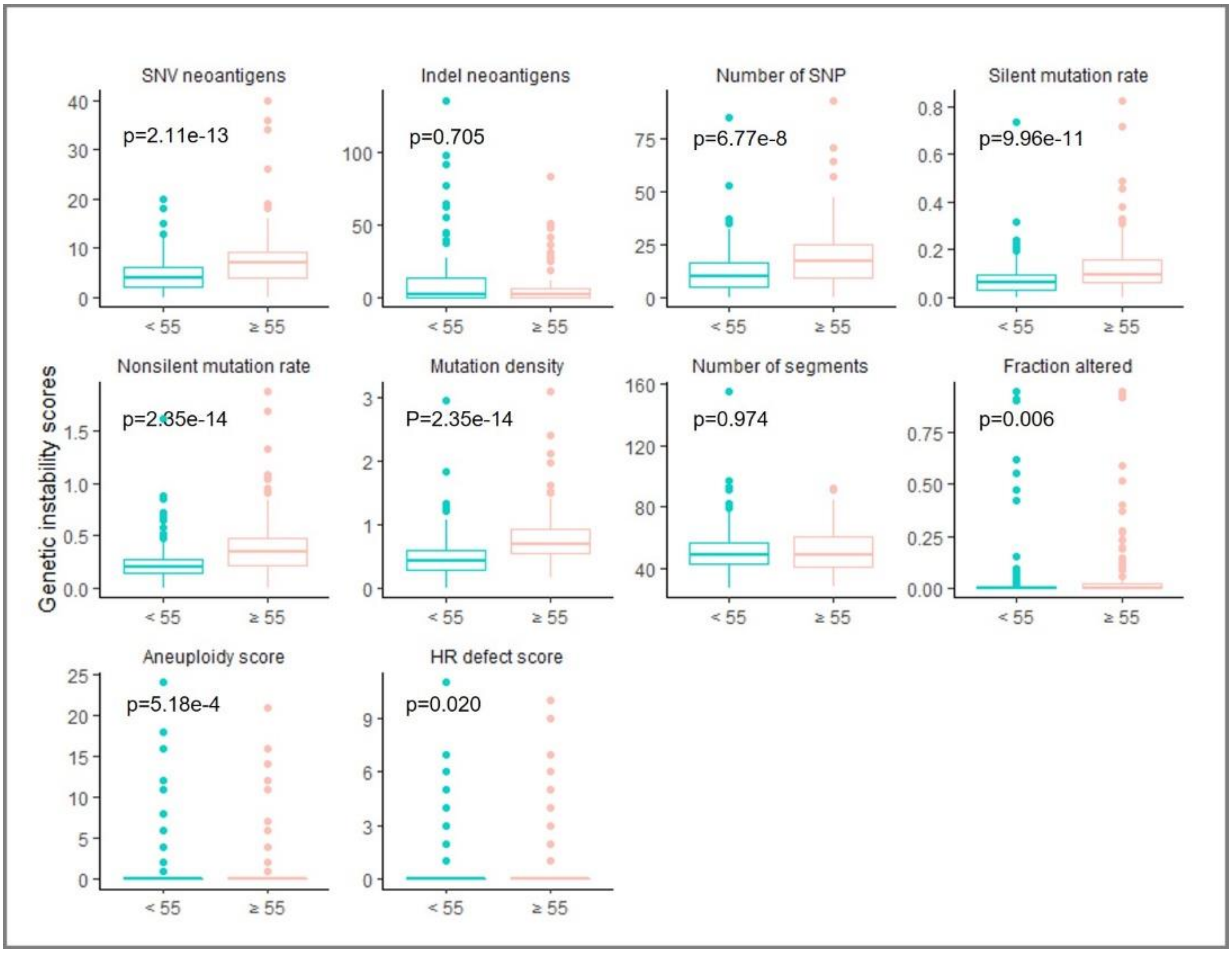

Supplementary Figure 4. Boxplots representing the distribution of 10 markers of genomic instability markers in thyroid tumors according toin samples from patients aged <55 and ≥55. SNV= Single Nucleotide Variant, SNP = Single Nucleotide Polymorphism, * Adjusted p-value < 0.05.

© 2022. Ruiz E et al. Published online at http://www.aginganddisease.org/EN/10.14336/AD.2022.1021

SUPPLEMENTARY DATA

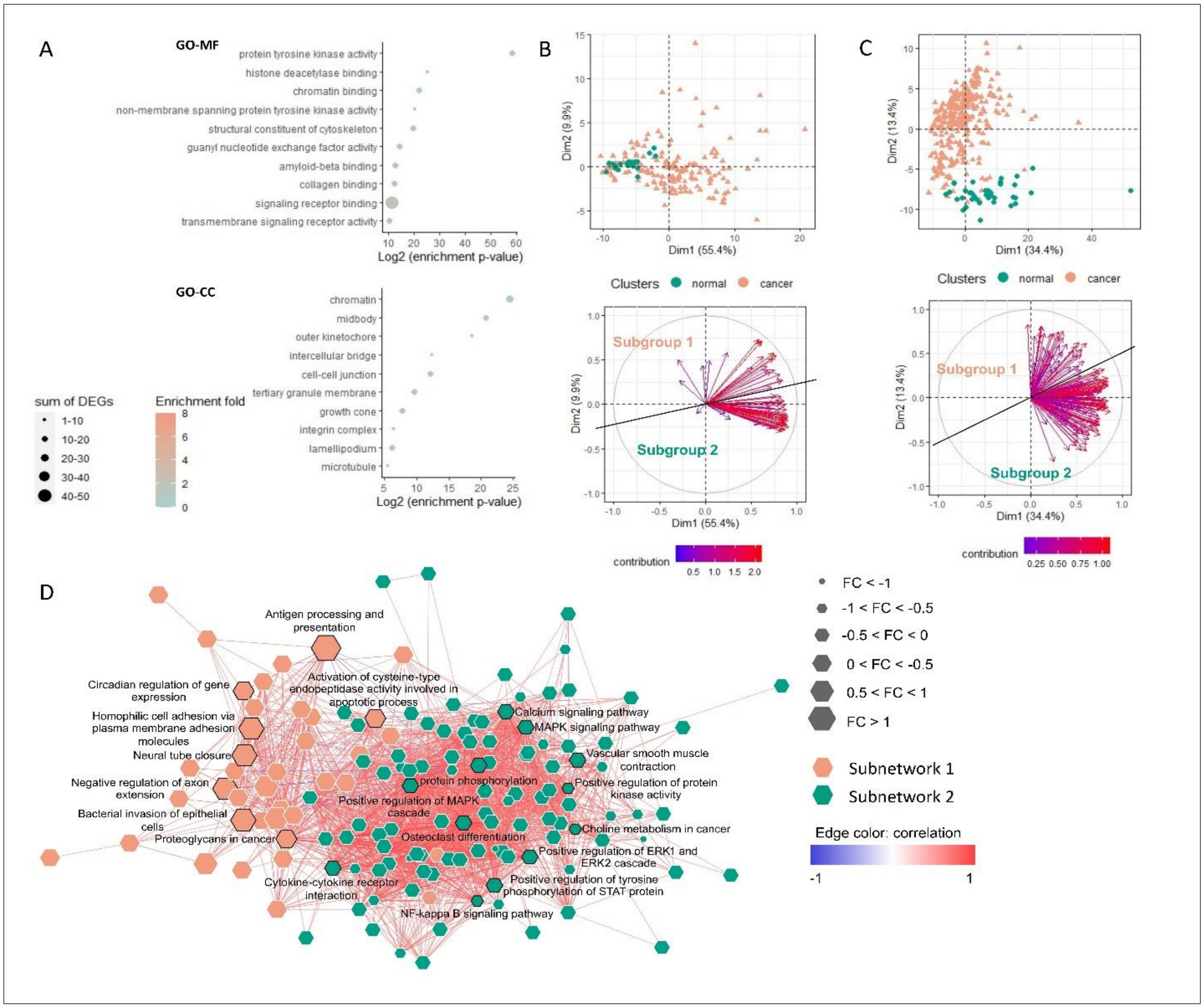

Supplementary Figure 5. Aging-induced thyroid transformation landscape. A. Dot plots representing the 10th first most significant GO-MF (top) and GO-CC (bottom) pathways selected after an enrichment analysis, according to their fold change adjusted p-value. Only pathways involving DEGs-AC and unique of tumor-aging or significantly different compared to the younger comparison were finally selected. **B-C.** PCA plots representing the heterogeneity of the aging samples (cancer and normal) for Old (**B**) and Young (**C**) cohorts - top) according to the significant pathways agglomerate z-cores (bottom). **D.** Network representing the spearman correlation between the enriched pathways in Young-cancer, with the node size depending on the pathway agglomerate z-score' fold change in Young-Cancer, compared to Young-normal samples. Only the KEGG and GO-BP entries with the highest contribution to the first two PCA were labeled

SUPPLEMENTARY DATA

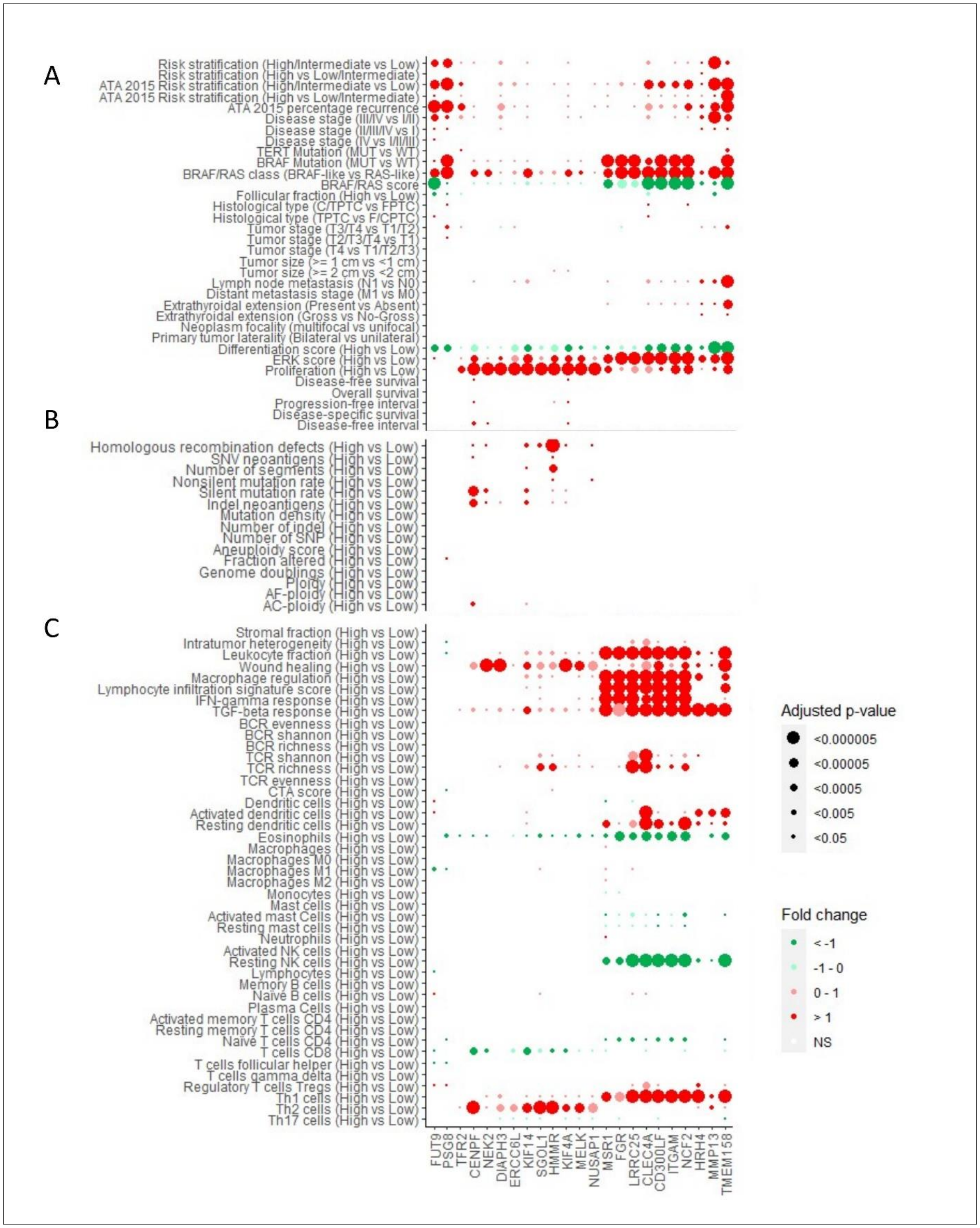

Supplementary Figure 6. Identification of a panel of 23 DEGs that are ageing-cancer specific and ageing-dependent associated with the clinical aggressive phenotype. Dot plots representing the DESeq2 parameters association analyses with estimation of Log2(fold change) and adjusted p-value for the 23 ageing-cancer specific - ageing dependent aggressive DEGs in the old tumor cohort – association with **(A)** clinical parameters, **(B)** genetic instability markers, and **(C)** immune cell infiltration and response. Adjusted Likelihood ratio test p-value, significant < 0.05.

© 2022. Ruiz E et al. Published online at http://www.aginganddisease.org/EN/10.14336/AD.2022.1021

SUPPLEMENTARY DATA

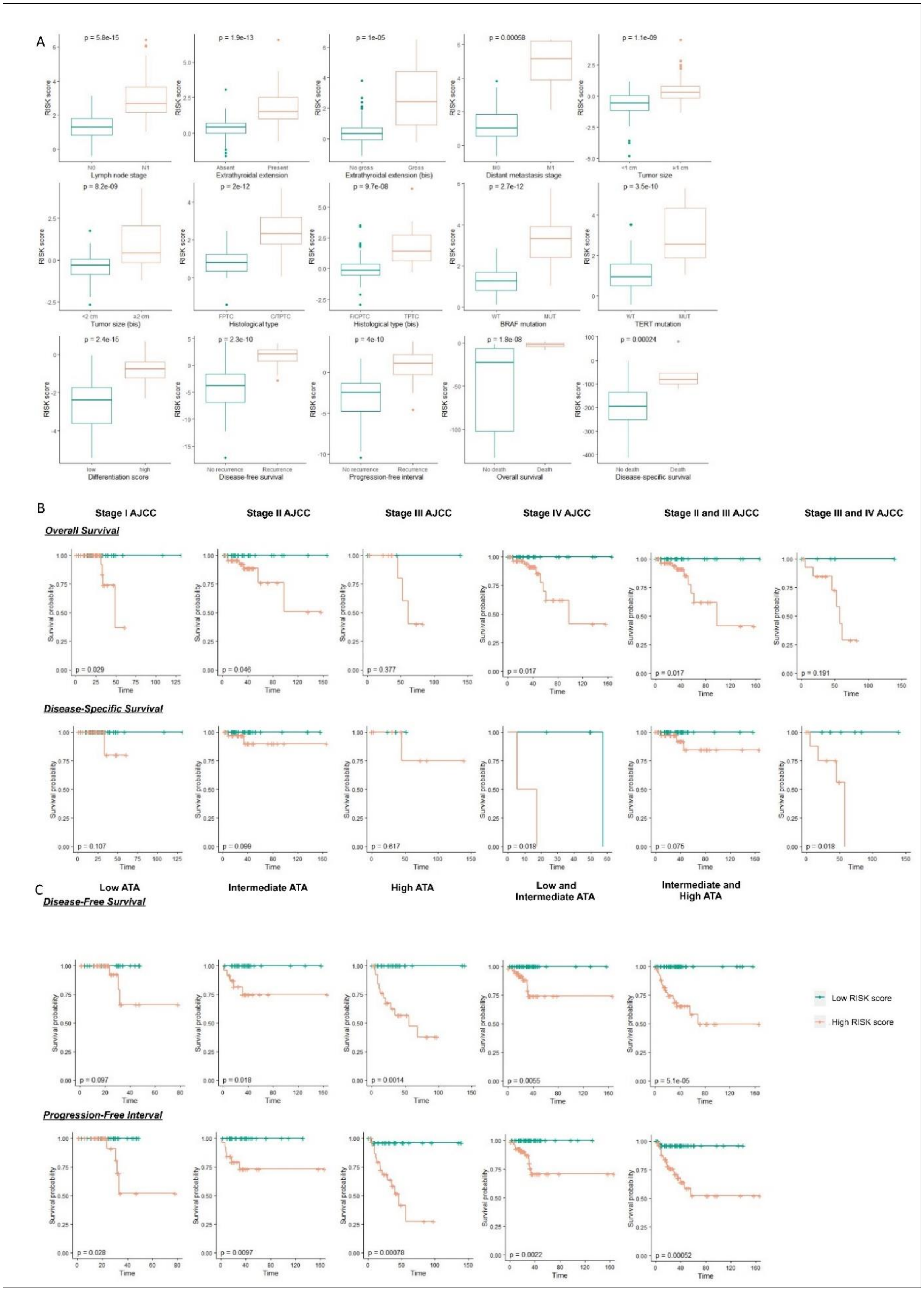

© 2022. Ruiz E et al. Published online at http://www.aginganddisease.org/EN/10.14336/AD.2022.1021

SUPPLEMENTARY DATA

Supplementary Figure 7. The 23-DEGs-AC panel a prognosis marker for ageing thyroid cancer patients. A. Box plots representing the distribution of the 23-DEGs-AC risk score according to clinical parameters (Kruskal-Wallis p-value). **B-C.** Kaplan Meier curves representing the survival probability according to the 23-DEGs-AC risk score (stratified according to the score mean – low, < mean; high, > mean) in specific aging subgroups defined by AJCC staging (**B**) or ATA risk stratification (**C**), for overall survival, and disease-specific survival (**B**) and disease-free survival and progression-free interval (**C**) (Log rank test p-value). P-value significant < 0.05.

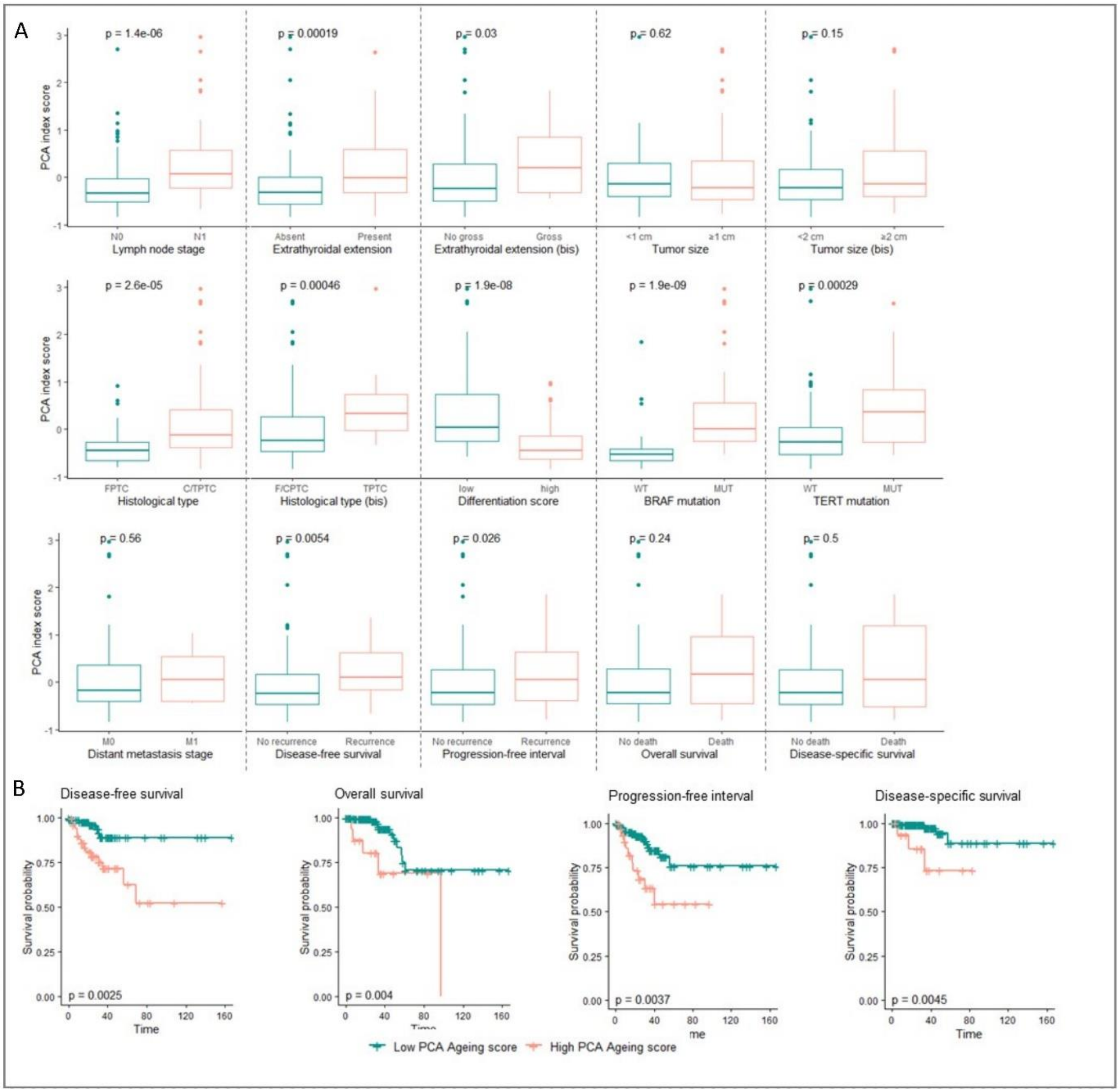

Supplementary Figure 8. The 23-DEGs-AC PCA aging score is a prognosis marker for ageing thyroid cancer patients. A. Box plots representing the distribution of the 23-DEGs-AC PCA aging score according to clinical parameters (Kruskal-Wallis p-value). **B**. Kaplan Meier curves representing the survival probability according to an optimized 23 DEGs-AC PCA aging score dependent stratification of old tumor samples for disease-free survival, progression-free interval, overall survival, and disease-specific survival (Log rank test p-value). P-value significant < 0.05.

© 2022. Ruiz E et al. Published online at http://www.aginganddisease.org/EN/10.14336/AD.2022.1021

SUPPLEMENTARY DATA

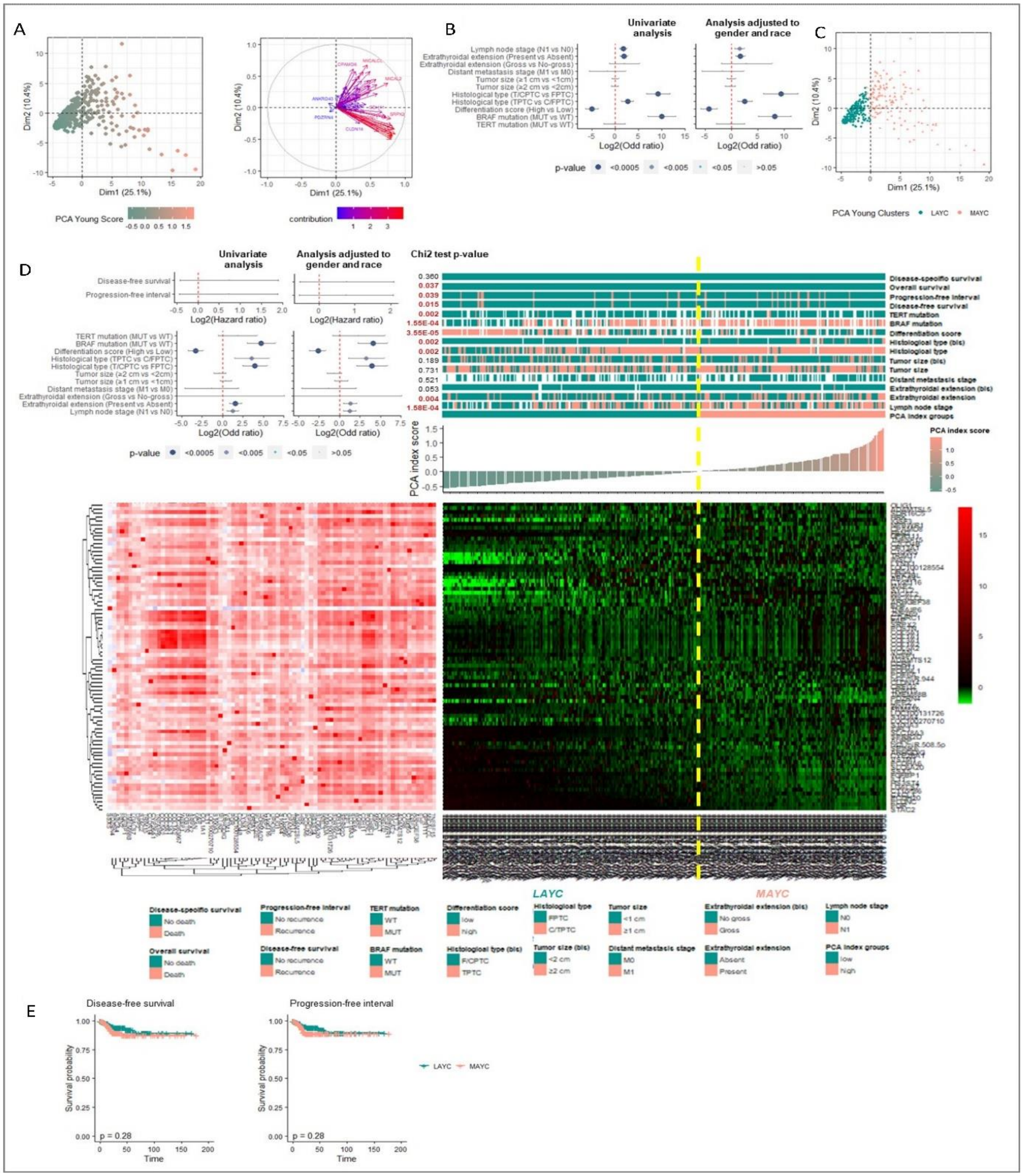

Supplementary Figure 9. Two young tumor clusters differentiated according to their aggressive phenotype. A. Principal component analysis illustrating the significance of the 64 DEGs-YC to explain the heterogeneity observed among the young tumor samples cohort (left) and their correlation (right). **B.** Logistic regression analyses between the 64-DEGs-YC PCA Young score and the aggressive clinical parameters, in a univariate and multivariate (adjusted to gender and race) analyses. **C.** Stratification of the young tumor cohort in 2 clusters based on the PCA Young score, LAYC (less aggressive young cluster) and MAYC (more aggressive Young cluster). **D.** The two clusters clinical association study by univariate and multivariate (adjusted to gender and race) logistic regression analyses of the clinical association of the 2 younger clusters (left top), with a heatmap representing the distribution of the gene expression and clinical parameters after a sorting according to the PCA Young score (Chi2 test p-value to evaluate the significant difference of the clinical parameters distribution according to the 2 young clusters) (right), and by a heatmap representing their Spearman correlation matrix in the younger tumor cohort (left bottom). **E.** Kaplan Meier curves representing the survival probability according to the two

© 2022. Ruiz E et al. Published online at http://www.aginganddisease.org/EN/10.14336/AD.2022.1021

younger tumor sample clusters for disease-free survival, progression-free interval, overall survival, and disease-specific survival (Log rank test p-value). P-values significant < 0.05

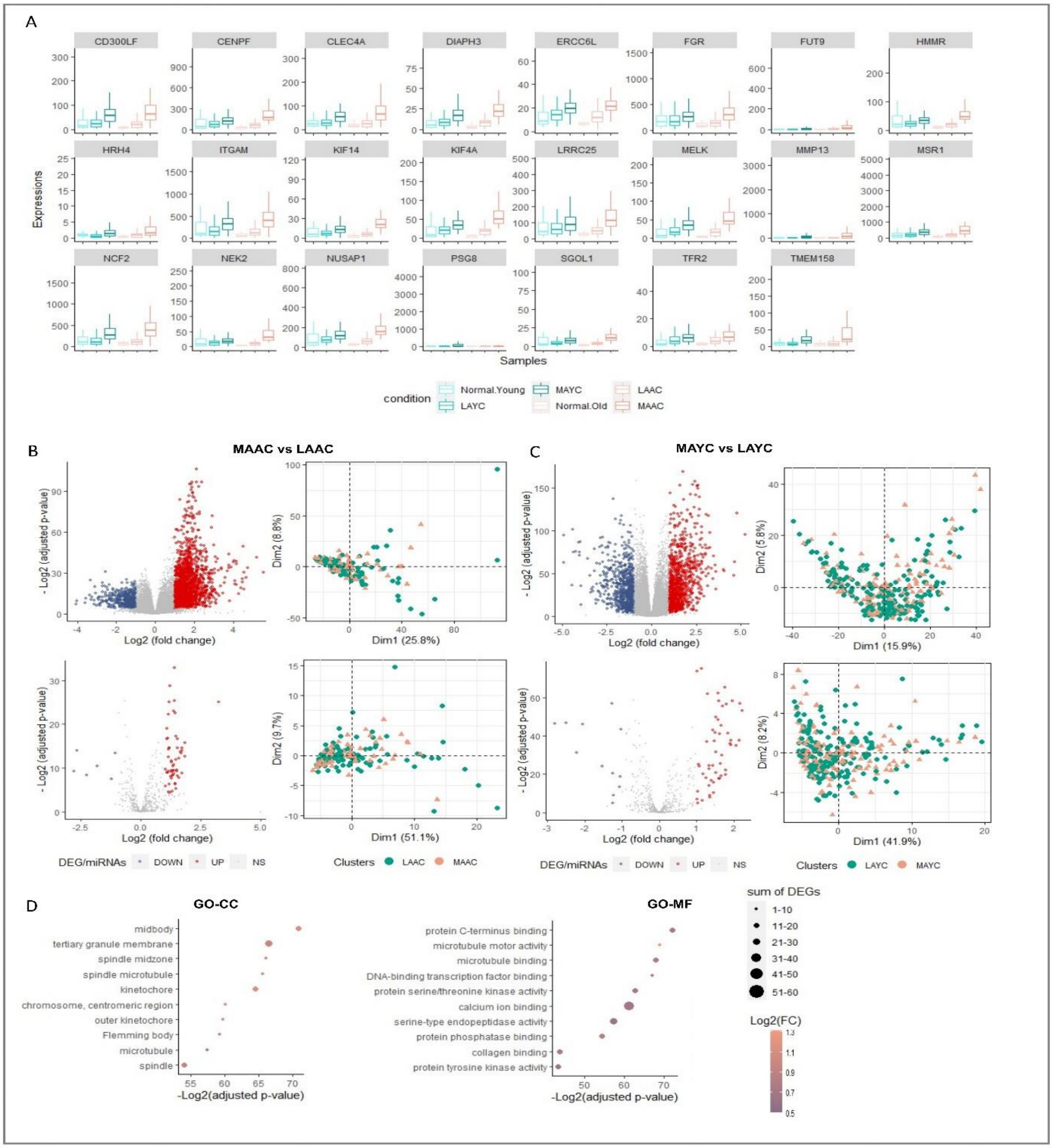

Supplementary Figure 10. The 23 DEGs-AC characterization across the thyroid samples. A. Boxplots representing the distribution of the 23-DEGs expression across the thyroid samples **B-C.** Volcano and corresponding Principal Component Analysis (PCA) plots of genes (left-top) and miRNAs (left-bottom) after Differential Expressed Genes (DEGs) analyses in the aging (**B**) and younger (**C**) cohorts (58 MAAC vs 103 LAAC – 419 and 1570 down and up-regulated genes and 6 and 56 down and up-regulated miRNAs in MAAC; 133 MAYC vs 188 LAYC – 669 and 883 down and up-regulated genes and 12 and 51 down and up-regulated miRNAs in MAYC). **D.** Dot plots representing the 10th first most significant GO-CC (left) and GO-MF (right) pathways selected after an enrichment analysis comparing MAAC vs LAAC, according to their fold change adjusted p-value. Only pathways unique of tumor-aging or significantly different compared to the younger comparison (MAYC vs LAYC) and including the 23-DEGs were finally selected.

© 2022. Ruiz E et al. Published online at http://www.aginganddisease.org/EN/10.14336/AD.2022.1021

SUPPLEMENTARY DATA

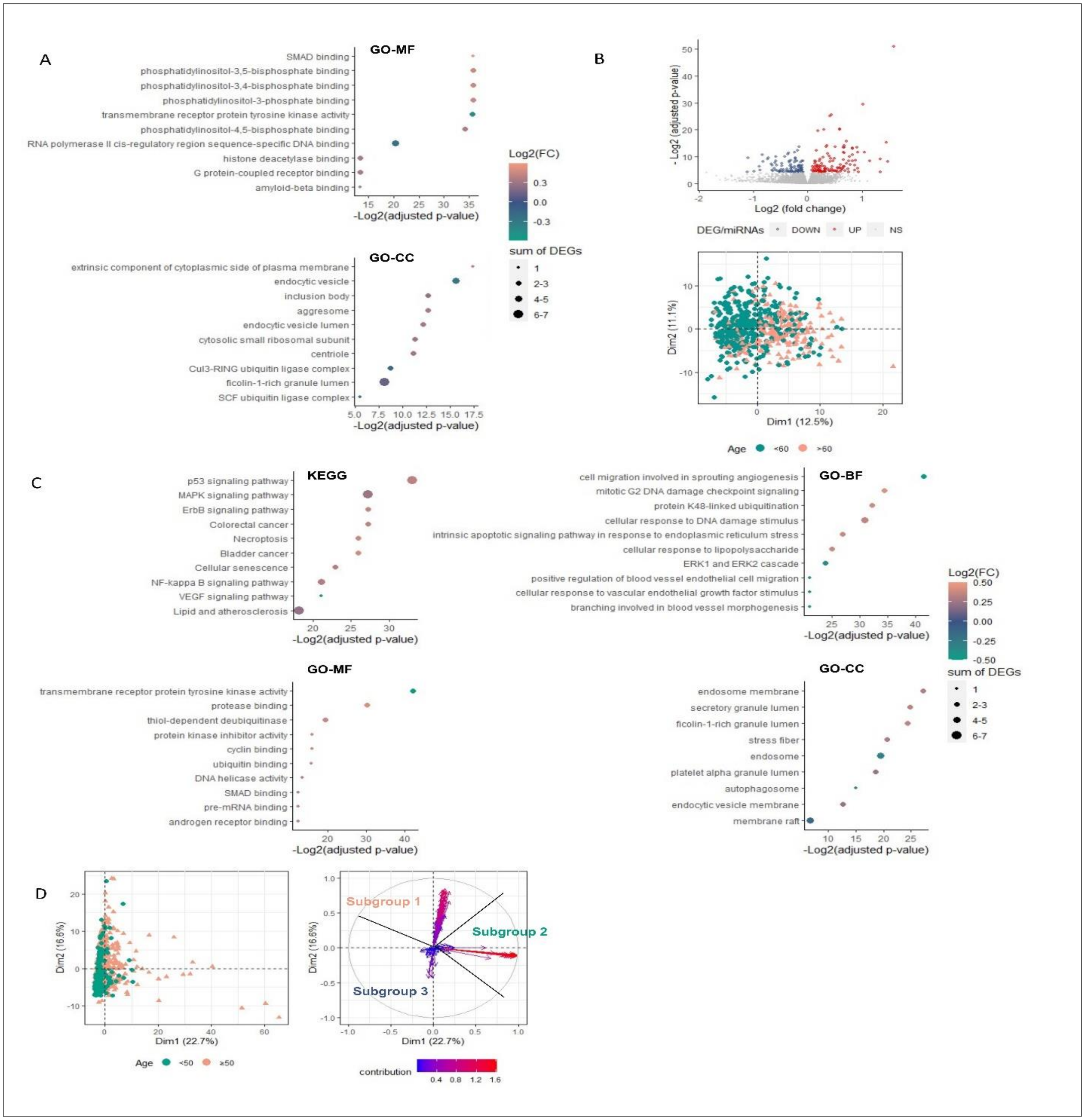

Supplementary Figure 11. Ageing related transcriptomic landscape in thyroid normal tissue. A. Dot plots representing the adjusted p-value of GO-MF (top) and GO-CC entries (bottom) differently enriched in the old (≥50) cohort, according to their fold change adjusted p-value. The 10 first most significant are described. **B.** Volcano (left) and corresponding Principal Component Analysis (PCA) plots of genes (right) after Differential Expressed Genes (DEGs) analyses in the aging normal cohort (≥60 vs <60, 234 old vs 445 samples– 89 and 143 down and up-regulated genes C. Dot plots representing the adjusted p-value of KEGG pathways (top-left), GO-BP (top-right), GO-MF (bottom-left), and GO-CC entries (bottom-right) differently enriched in the old (≥60) cohort, according to their fold change adjusted p-value. The 10 first most significant are described. **D.** PCA plots representing the heterogeneity of the samples (Aging vs Young normal samples ≥50 vs <50 - left) according to the significant pathways agglomerate z-scores (right). 3 subgroups of pathways were identified.

© 2022. Ruiz E et al. Published online at http://www.aginganddisease.org/EN/10.14336/AD.2022.1021

SUPPLEMENTARY DATA

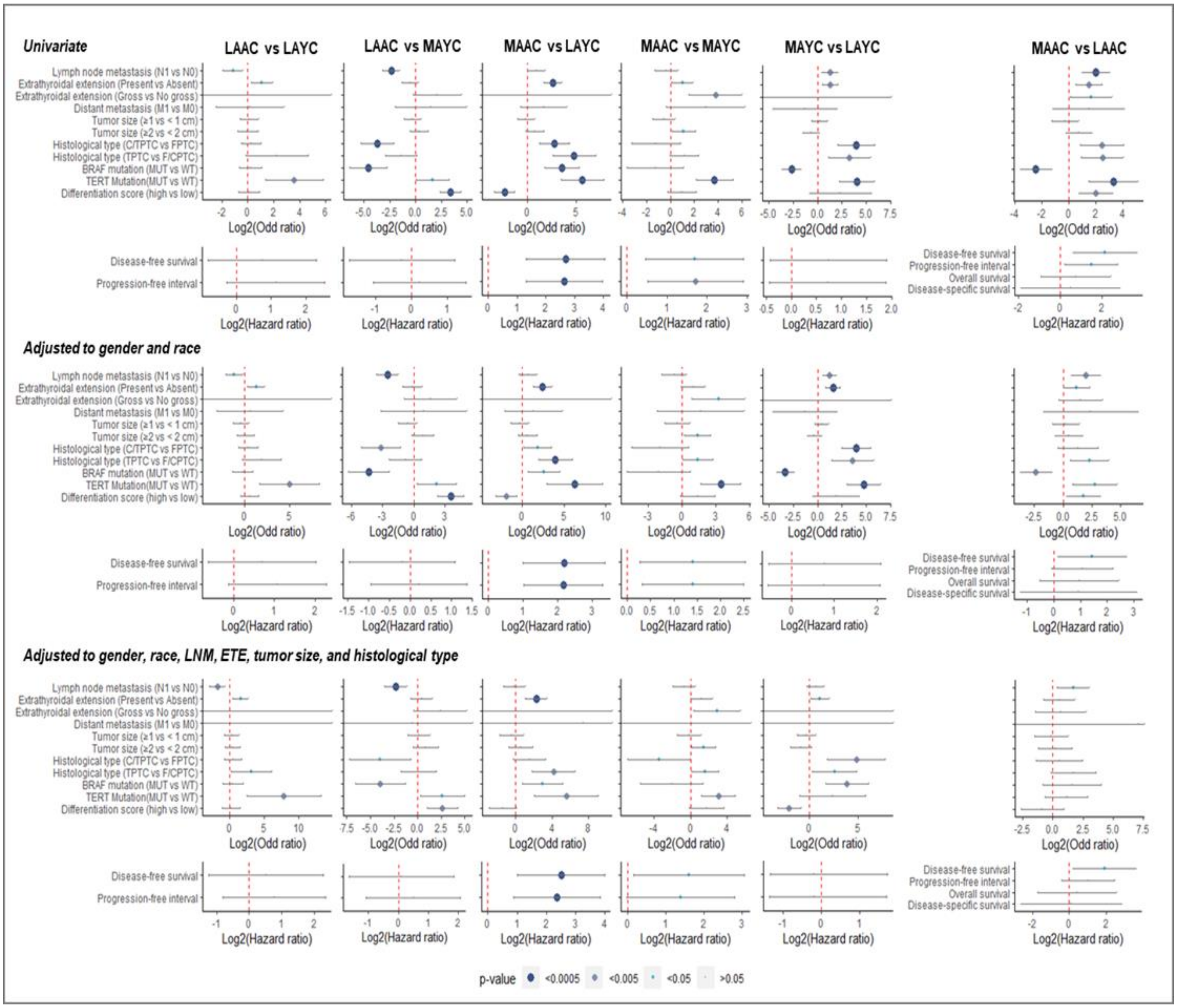

Supplementary Figure 12. Clinical and outcome comparison among the 4 thyroid tumor clusters. Plots showing univariate (top) and multivariate logistic regression analyses (adjusted to gender and race (middle) and adjusted to gender, race, LNM, ETE, tumor size, and histological type (bottom) testing, from left to right, LAAC vs LAYC, LAAC vs MAYC, MAAC vs LAYC, MAAC vs MAYC, MAYC vs LAYC, and MAAC vs LAAC. OS and DSS Cox regression analyses were not shown for LAYC and MAYC as not death event was present in these clusters. CPTC, Classical variant papillary thyroid carcinoma (PTC); ETE, extrathyroidal extension; FPTC, Follicular variant PTC; LNM, Lymph node metastasis; MUT, Mutant; TPTC, Tall-cell variant PTC; WT, Wild-type. p-value significant < 0.05.

© 2022. Ruiz E et al. Published online at http://www.aginganddisease.org/EN/10.14336/AD.2022.1021

SUPPLEMENTARY DATA

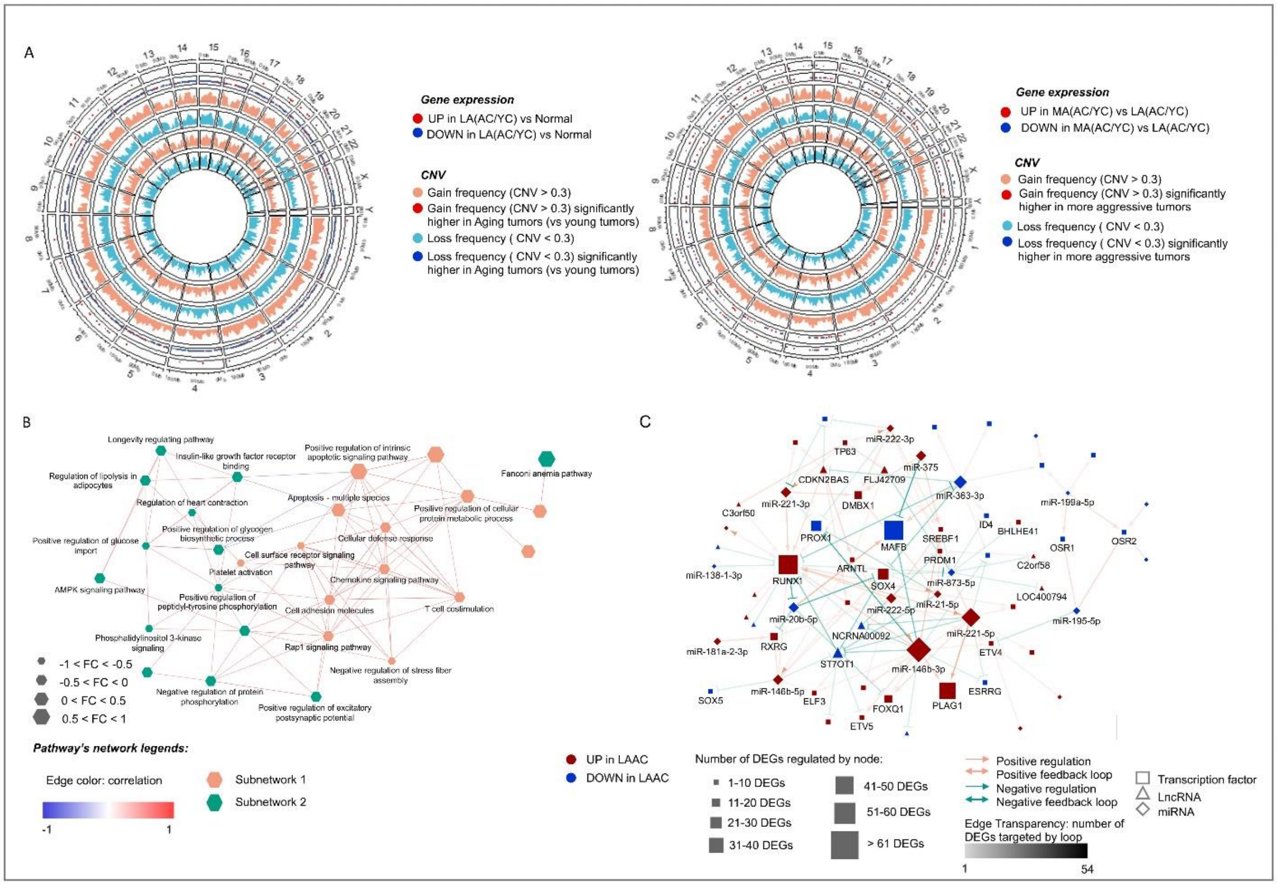

Supplementary Figure 13. Molecular landscape in the aging cancer models. A. Circular plots representing (from outer to inner) Log2 fold change of the DEG/miRNAs expression between less aggressive tumors vs normal samples (left), and more aggressive vs less aggressive clusters (right), and their corresponding frequency of gene copy number gain (CNV > 0.3) or loss (CNV < 0.3) for LAAC and LAYC (left), and MAAC and MAYC (right). **B.** Network representing the spearman correlation between the enriched pathways in LAAC samples, with the node size depending on the pathway agglomerate z-score' fold change in LAAC compared to Aging Normal samples. Two clusters were identified through a PCA computing the pathways' agglomerate scores among the samples. Only the KEGG and GO-BP entries with the highest contribution to the first two PCA were labeled. **C.** Network representing the loops between TFs, miRNAs and LncRNAs, strongly regulating the LAAC specific DEGs. Only TF/miRNA/DEG, TF/LncRNA/DEG, or miRNA/LncRNA/DEG loops with spearman correlation > |0.5| were selected.

SUPPLEMENTARY DATA

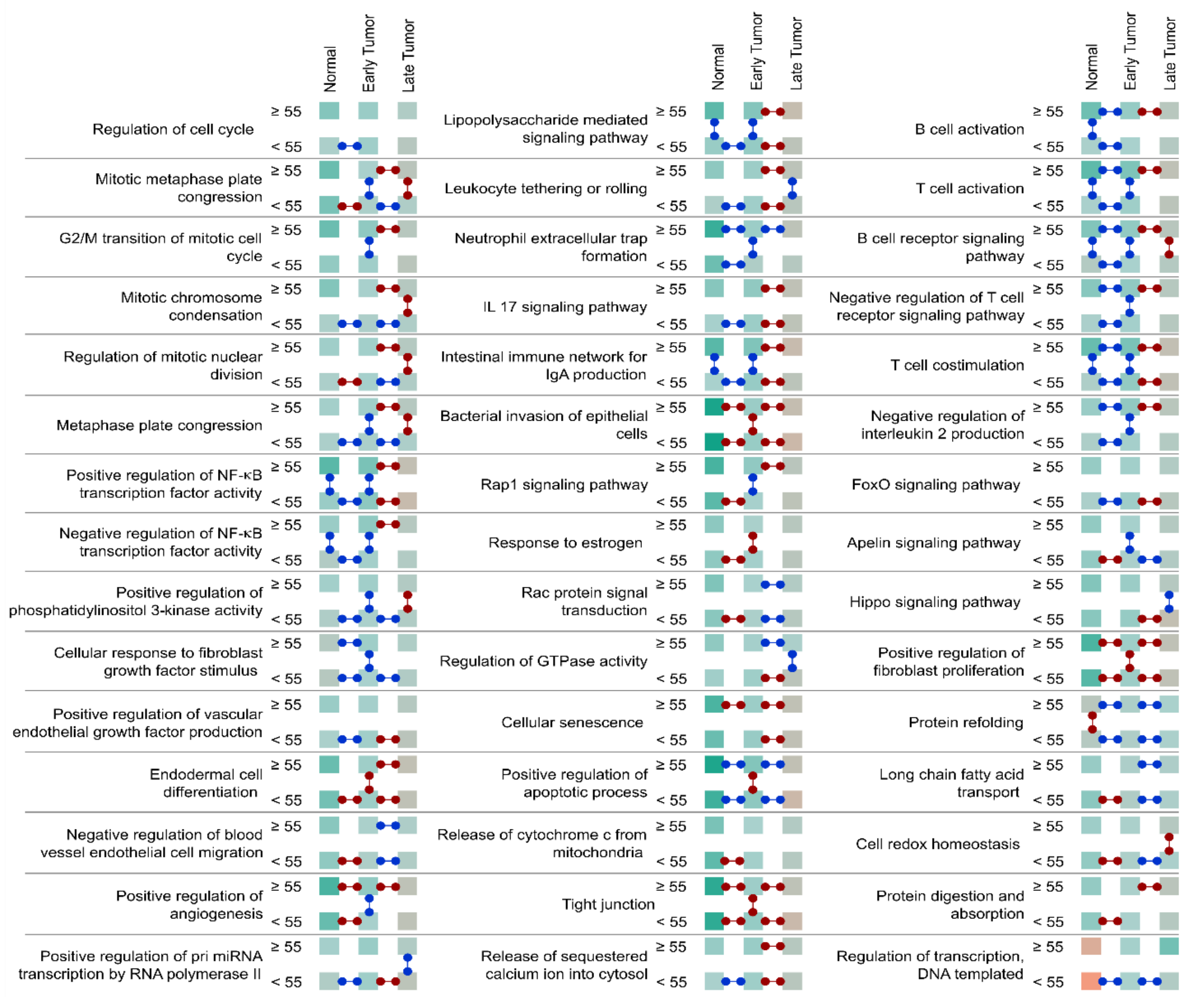

Supplementary Figure 14. The global aging landscape. Heatmap representing the mean of significant pathway NES score enriched pathways and processes in tumor stage comparisons. Arrows represent a significant FC (red, positive; blue, negative).

SUPPLEMENTARY DATA

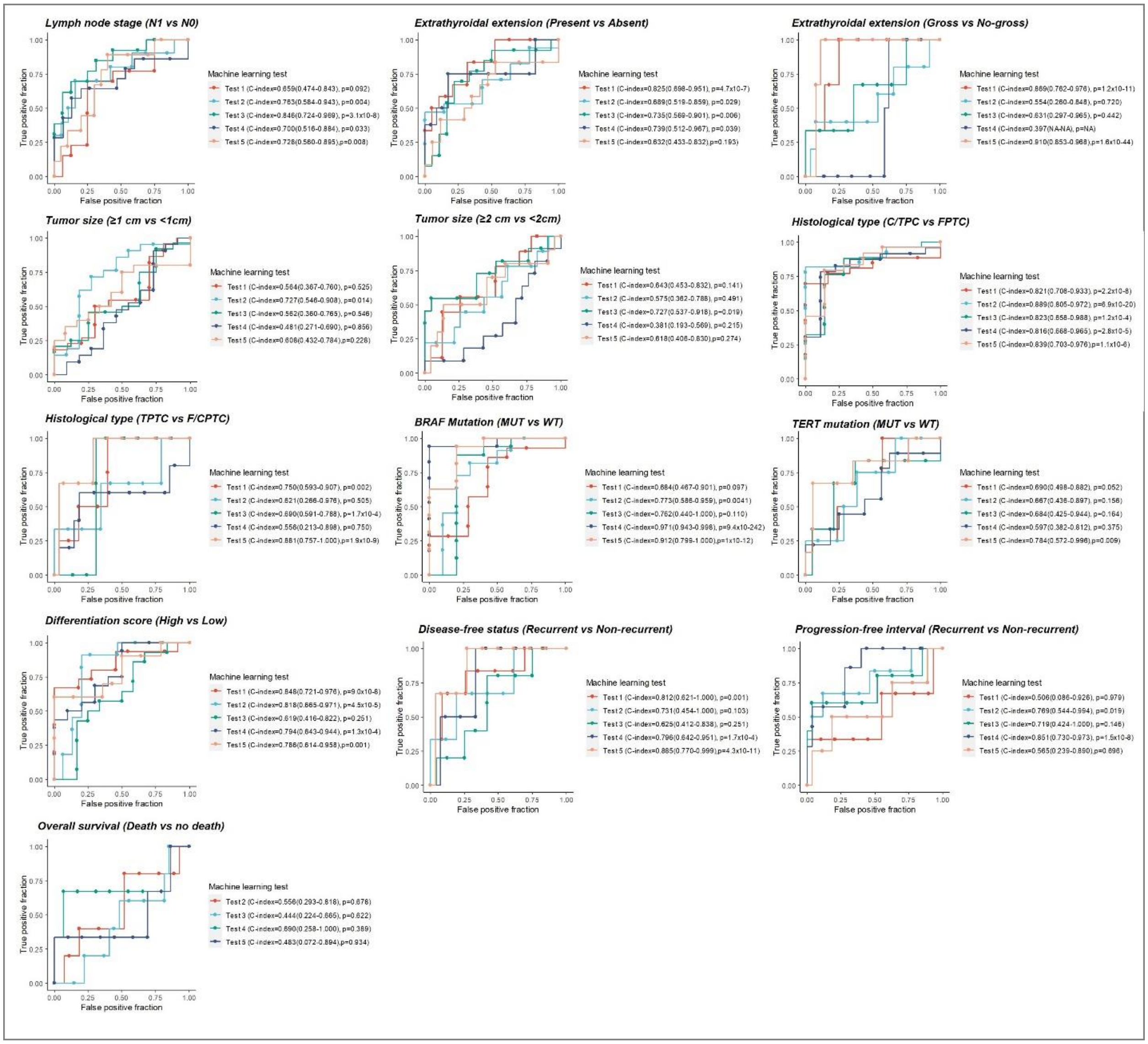

Supplementary Figure 15. 23-DEGs panel prognostication performance in the in the old cancer TCGA cohort in a five-fold machine learning methodology. Receiver operative characteristics curves of the 23-DEGs panel for prediction of aggressive parameters, across the old cancer cohort partitioned in 5 parts in order to run machine learning linear discriminant analyses. Area under curve estimated with concordance indexes (C-indexes).

© 2022. Ruiz E et al. Published online at http://www.aginganddisease.org/EN/10.14336/AD.2022.1021

SUPPLEMENTARY DATA

A

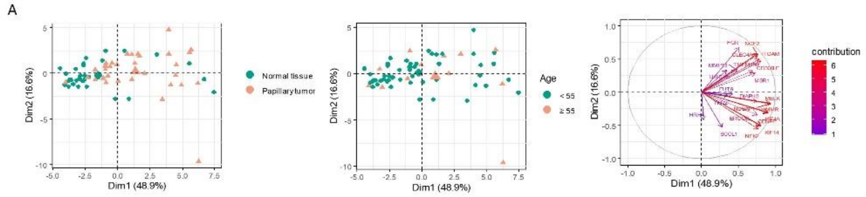

B

Population	Parameter analyzed	RISK score: Cut-off	Sensitivity	Specificity	PPV	NPV	AUC	OR (CI-95%-OR)	OR p-value	Kruskal test p-value	PCA index score: OR (CI-95%-OR)	OR p-value	Kruskal test p-value
PTC + normal samples	Malignancy	0.48	0.94	1.00	1.00	0.94	0.994 (0.983-1.005)	121.31 (1.82-8091.99)	$3.14x10^{-16}$	$1.70x10^{-11}$	4.48 (1.74-11.54)	$3.73x10^{-4}$	$4.63x10^{-5}$
	Age (≥55 vs < 55)	0.19	0.83	0.84	0.56	0.96	0.904 (0.817-0.991)	6.71 (2.24-20.15)	$4.68x10^{-7}$	$1.54x10^{-5}$	1.81 (0.75-4.37)	0.186	0.172
PTC	Age (≥55 vs < 55)	0.79	1.00	1.00	1.00	1.00	1 (1-1)	$4.4x10^{58}$ (0.00-Inf)	$5.22x10^{-8}$	$6.14x10^{-5}$	0.23 (0.02-2.48)	0.161	0.283
	Tumor stage (T3/T4 vs T1/T2)	0.80	1.00	1.00	1.00	1.00	1 (1-1)	$1x10^{18}$ (0.00-Inf)	$1.98x10^{-8}$	$2.94x10^{-5}$	7677.78 ($5.2x10^{-5}$-$1.1x10^{12}$)	0.050	0.118
	Tumor stage (T2/T3/T4 vs T1)	0.55	1.00	1.00	1.00	1.00	1 (1-1)	$2.1x10^{49}$ (0.00-Inf)	$2.74x10^{-8}$	$1.66x10^{-4}$	1.00 (0.22-4.57)	0.998	0.961
	Tumor stage (T4 vs T1/T2/T3)	1.00	1.00	1.00	1.00	1.00	1 (1-1)	22.51 (0.00-Inf)	$1.10x10^{-4}$	0.020	1.34 (0.41-4.36)	0.630	0.484
	Tumor stage (III/IV vs I)	0.07	1.00	0.89	0.87	1.00	0.988 (0.963-1.012)	21.23 (1.86-242.43)	$5.51x10^{-9}$	$3.77x10^{-6}$	1.79 (0.56-5.73)	0.321	0.329
	Tumor stage (IV vs III)	0.94	1.00	1.00	1.00	1.00	1 (1-1)	$1.6x10^{10}$ (0.00-Inf)	$8.11x10^{-6}$	0.005	0.44 (0.047-4.16)	0.458	0.789
	Histological type (F/CPTC vs Others)	0.33	1.00	0.96	0.83	1.00	0.993 (0.972-1.013)	71.86 (0.25-20656.16)	$1.34x10^{-6}$	0.001	1.49 (0.47-4.78)	0.497	0.512
	Histological type (FPTC vs Others)	0.35	0.96	1.00	1.00	0.86	0.994 (0.976-1.011)	$2.9x10^{10}$ ($6.2x10^{-10}$-$1.4x10^{30}$)	$2.16x10^{-7}$	$2.01x10^{-4}$	0.74 (0.20-2.71)	0.648	0.761
	Tumor size (≥ 1 vs < 1 cm)	1.00	1.00	1.00	1.00	1.00	1 (NaN-NaN)	2166.51 (0.00-Inf)	0.003	0.094	0.62 (0.15-2.62)	0.512	0.469
	Tumor size (≥ 2 vs < 2 cm)	1.00	1.00	1.00	1.00	1.00	1 (1-1)	$4x10^{9}$ (0.00-Inf)	0.000	$2.10x10^{-6}$	Inf (0.00-Inf)	$1.10x10^{-4}$	0.020
	LN Metastases size (≥ 1 vs < 1 cm)	0.98	0.71	1.00	1.00	0.43	0.905 (0.701-1.109)	7.30 (0.66-80.64)	0.003	0.032	1.65 (0.51-5.30)	0.398	0.388
	LN Metastases size (≥ 2 vs < 2 cm)	0.80	1.00	0.86	0.91	1.00	0.886 (0.659-1.113)	7.26 (1.25-42.09)	$3.19x10^{-4}$	0.008	7.26 (0.60-88.37)	0.072	0.075
	Capsular effraction (yes vs no)	0.43	1.00	0.80	0.93	1.00	0.862 (0.585-1.138)	4.45 (1.26-15.79)	0.002	0.021	1.40 (0.30-6.65)	0.671	0.452
	Extrathyroidal extension (yes vs no)	0.80	1.00	1.00	1.00	1.00	1 (1-1)	$1.2x10^{35}$ (0.00-Inf)	$7.67x10^{-11}$	$3.00x10^{-6}$	1.97 (0.42-9.30)	0.379	0.288
	lymphocyte reaction (yes vs no)	0.96	1.00	1.00	1.00	1.00	1 (1-1)	$4.6x10^{12}$ (0.00-Inf)	$1.55x10^{-11}$	1.06E-06	0.59 (0.019-18.43)	0.762	0.576
	vascular invasion (yes vs no)	1.00	1.00	1.00	1.00	1.00	1 (1-1)	$2.86x10^{8}$ (0.00-Inf)	$9.00x10^{-6}$	0.005	0.79 (0.23-2.70)	0.705	0.502
	BRAF mutation status(MUT vs WT)	0.75	0.95	1.00	1.00	0.86	0.992 (0.969-1.015)	84.27 (0.10-68863.77)	$1.40x10^{-6}$	$3.30x10^{-4}$	1.30 (0.19-8.69)	0.789	0.801
	NRAS mutation status (MUT vs WT)	1.00	1.00	1.00	1.00	1.00	1 (NaN-NaN)	3.87 (0.00-Inf)	0.004	0.096	0.64 (0.15-2.85)	0.558	0.380
	RET-PTC1 mutation status(MUT vs WT)	0.50	1.00	1.00	1.00	1.00	1 (1-1)	$2.4x10^{56}$ (0.00-Inf)	$4.52x10^{-7}$	0.001	1.40 (0.26-7.56)	0.689	0.761
	Lymph node stage (N1 vs N0)	0.66	0.95	1.00	1.00	0.93	0.996 (0.986-1.007)	223.21 (0.15-327658.86)	$1.52x10^{-10}$	$1.52x10^{-6}$	2456.41 ($1.9x10^{-4}$-$3.1x10^{10}$)	0.047	0.125
	Distant metastasis stage (M1 vs Mx)	1.00	1.00	1.00	1.00	1.00	1 (NaN-NaN)	5.48 (0.00-Inf)	0.003	0.094	0.23 (0.02-2.48)	0.161	0.283
PTC (age ≥55)	Tumor stage (T3/T4 vs T1/T2)	0.57	1.00	1.00	1.00	1.00	1 (1-1)	$5.6x10^{34}$ (0.00-Inf)	0.006	0.064	54.06 ($6.8x10^{-5}$-$4.3x10^{7}$)	0.322	0.480
	Tumor stage (T2/T3/T4 vs T1)	0.57	1.00	1.00	1.00	1.00	1 (1-1)	$5.6x10^{34}$ (0.00-Inf)	0.006	0.064	0.063 ($1.1x10^{-5}$-356.70)	0.372	1.000
	Tumor stage (T4 vs T1/T2/T3)	1.00	1.00	1.00	1.00	1.00	1 (1-1)	25400.03 (0.00-Inf)	0.006	0.064	1.15 (0.045-29.40)	0.931	1.000
	Tumor stage (III/IV vs I)	0.86	1.00	1.00	1.00	1.00	1 (NaN-NaN)	$8.1x10^{12}$ (0.00-Inf)	0.020	0.143	2.37 (0.064-87.19)	0.635	0.699
	Tumor stage (IV vs III)	0.34	1.00	0.67	0.75	1.00	0.889 (0.581-1.197)	25.07 (0.057-11000.15)	0.085	0.127	0.00 (0.00-Inf)	0.020	0.143
	Histological type (F/CPTC vs Others)	1.00	1.00	1.00	1.00	1.00	1 (NaN-NaN)	370862.27 (0.00-Inf)	0.017	0.134	7.21 (0.091-568.88)	0.326	0.699
	Histological type (FPTC vs Others)	0.70	1.00	1.00	1.00	1.00	1 (NaN-NaN)	$6.9x10^{23}$ (0.00-Inf)	0.017	0.134	0.76 (0.024-24.44)	0.878	1.000
	Tumor size (≥ 1 vs < 1 cm)	0.57	1.00	1.00	1.00	1.00	1 (NaN-NaN)	$5.3x10^{154}$ (0.00-Inf)	0.020	0.143	0.76 (0.024-24.44)	0.878	1.000
	Tumor size (≥ 2 vs < 2 cm)	0.22	1.00	0.75	0.67	1.00	0.875 (0.529-1.221)	4513.27 ($1x10^{-10}$-$2x10^{17}$)	0.061	0.165	Inf (0.00-Inf)	0.006	0.064
	LN Metastases size (≥ 1 vs < 1 cm)	0.83	0.67	1.00	1.00	0.50	0.667 (NaN-NaN)	8.10 (0.033-1969.56)	0.301	0.655	7.80 (0.025-2431.08)	0.418	0.770
	LN Metastases size (≥ 2 vs < 2 cm)	0.83	0.67	1.00	1.00	0.50	0.667 (NaN-NaN)	8.10 (0.033-1969.56)	0.301	0.655	4.03 (0.13-128.66)	0.416	0.275
	Capsular effraction (yes vs no)	0.66	1.00	1.00	1.00	1.00	1 (NaN-NaN)	$6.9x10^{78}$ (0.00-Inf)	0.025	0.157	125.12 ($1.5x10^{-4}$-$1x10^{8}$)	0.196	0.317
	Extrathyroidal extension (yes vs no)	0.31	1.00	1.00	1.00	1.00	1 (1-1)	$5.1x10^{171}$ (0.00-Inf)	0.002	0.034	8.77 (0.014-5622.51)	0.436	0.617
	lymphocyte reaction (yes vs no)	0.35	1.00	0.80	0.67	1.00	0.9 (0.623-1.177)	9.22 (0.23-366.89)	0.056	0.121	2.21 (0.0091-537.25)	0.769	0.770
	vascular invasion (yes vs no)	0.59	1.00	1.00	1.00	1.00	1 (NaN-NaN)	$1.8x10^{63}$ (0.00-Inf)	0.020	0.143	2.23 (0.043-115.11)	0.689	0.643
	BRAF mutation status(MUT vs WT)	0.73	0.67	1.00	1.00	0.67	0.833 (0.371-1.295)	20.07 (0.02-19969.19)	0.087	0.248	Inf (0.00-Inf)	0.034	0.180
	NRAS mutation status (MUT vs WT)	1.00	1.00	1.00	1.00	1.00	1 (NaN-NaN)	$8.36x10^{6}$ (0.00-Inf)	0.025	0.157	Inf (0.00-Inf)	0.034	0.180
	RET-PTC1 mutation status(MUT vs WT)	0.67	1.00	1.00	1.00	1.00	1 (NaN-NaN)	$3.3x10^{24}$ (0.00-Inf)	0.025	0.157	0.36 (0.009-14.70)	0.587	0.564
	Lymph node stage (N1 vs N0)	0.39	1.00	1.00	1.00	1.00	1 (1-1)	$5x10^{80}$ (0.00-Inf)	0.004	0.053	64.63 (0.0032-$1.30x10^{6}$)	0.182	0.480
	Distant metastasis stage (M1 vs Mx)	0.98	1.00	1.00	1.00	1.00	1 (NaN-NaN)	$2x10^{10}$ (0.00-Inf)	0.025	0.157	0.20 (0.00063-61.77)	0.532	1.000

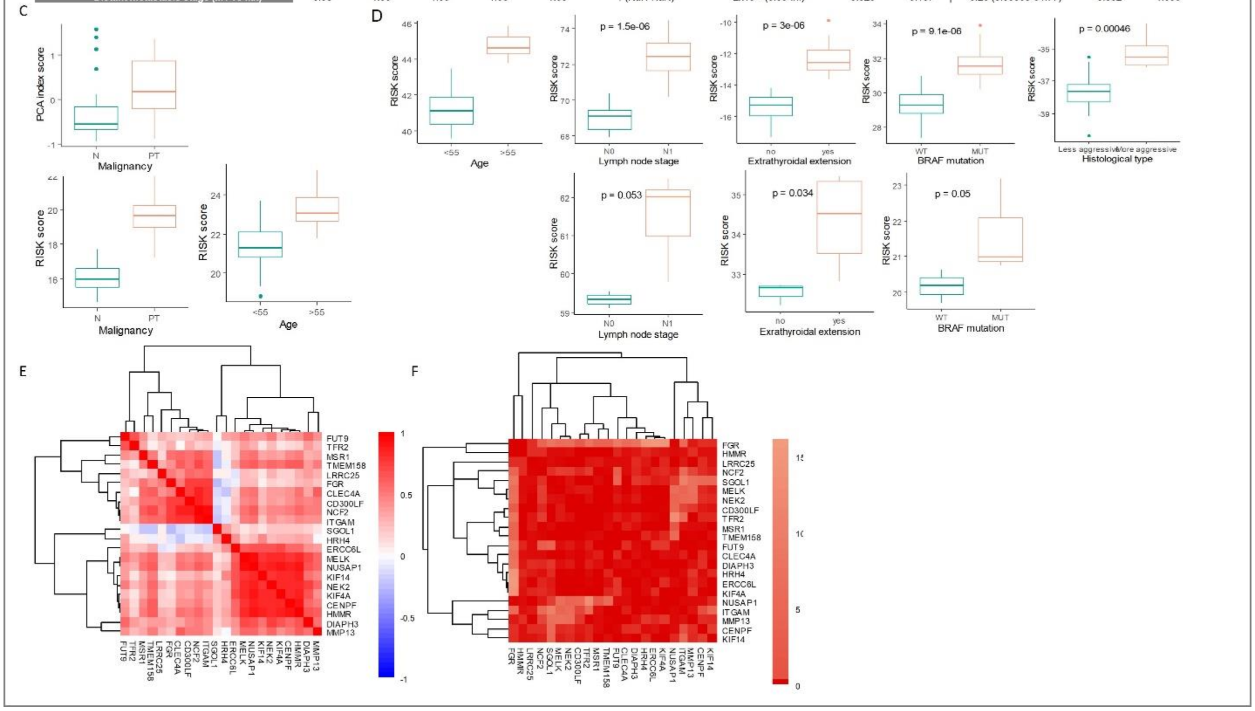

Supplementary Figure 16. 23-DEGs panel diagnostic and prognostication performance in the GSE60542 dataset. Dataset contains 33 PTCs and 30 samples. **A.** Principal component analysis on thyroid samples according to 22 AC-DEGs (*PSG8* not included in the GPL570 Affymetrix platform), samples stratified according to the samples' malignancy (left) and age (middle), and 22 DEGs highly correlated (right). **B.** Diagnostic and prognostication performance of the 22-DEGs panel tested with the risk score ROC curve and logistic regression (left) and PCA index score linear regression analyses (right), for all samples, the PTC samples, and the old PTC samples. Regression likelihood ratio p-value and Kruskal-Wallis p-value for corresponding distribution analysis were estimated because of the regression model convergence leading to OR calculation inaccuracy. p-value significant < 0.05. **C.** Boxplots representing the PCA-Index score distribution according to malignancy in all samples (top), and the 22-Risk score distribution according to malignancy

© 2022. Ruiz E et al. Published online at http://www.aginganddisease.org/EN/10.14336/AD.2022.1021

and age in all samples (bottom). D. Boxplots representing the 22-Risk score distribution according to age, lymph node stage, extrathyroidal extension, *BRAF*^{V600E} mutation, and histological type, in the PTC (top) and in the old PTC (bottom) cohorts. **E.** Spearman correlation matrix of the 22 DEGs in the PTC cohort. **F.** heatmap representing the adjusted p-values comparing the GSE60542 22-DEG panel correlation matrix with the TCGA 22-DEGG panel correlation matrix. Redder is the p-value and less the comparison is significant showing that the 22 DEGs behave similarly in the GSE60542 dataset as in the TCGA old cohort. CI-95%, 95% confidence interval; CPTC, classic (papillary) TC; FPTC, follicular-variant PTC; MUT, mutant; NPV, Negative predictive value; OR, Odd ratio; PPV, Positive predictive value; WT, wild-type.

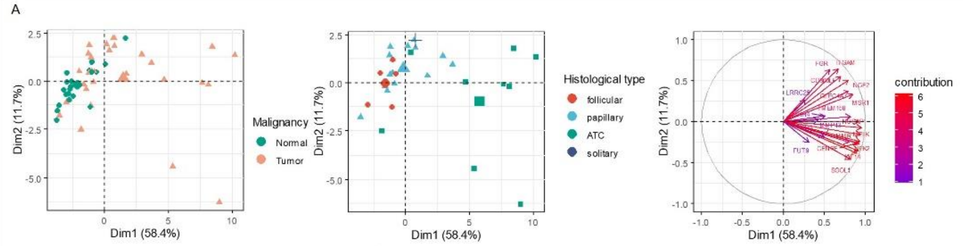

B

		All samples		All samples except ATC		Only PTC								
		Malignancy (tumor vs normal)	Age (≥55 vs <55)	Malignancy (tumor vs normal)	Age (≥55 vs <55)	Lymph node metastasis (yes vs no)	Extrathyroidal extension (yes vs no)	Capsule invasion (yes vs no)	BRAF mutation (MUT vs WT)	Histological type (CPTC vs FPTC)	Tumor size (≥1 cm vs <1cm)	Tumor size (≥2 cm vs <2cm)
RISK score	Cut-off	0.73	0.98	0.59	0.98	0.72	1.00	0.96	0.35	0.73	1.00	1.00
	Sensitivity	0.93	1.00	0.95	1.00	0.89	1.00	0.86	1.00	1.00	1.00	0.61
	Specificity	0.95	1.00	1.00	1.00	1.00	1.00	1.00	0.90	1.00	1.00	1.00
	PPV	0.96	1.00	1.00	1.00	1.00	1.00	1.00	0.89	1.00	1.00	1.00
	NPV	0.90	1.00	0.95	1.00	0.90	1.00	0.93	1.00	1.00	1.00	0.36
	AUC	0.981 (0.954, 1.008)	1 (1, 1)	0.988 (0.961, 1.014)	1 (1, 1)	0.975 (0.917, 1.033)	1 (1, 1)	0.978 (0.926, 1.03)	0.988 (0.953, 1.022)	1 (1, 1)	1 (1, 1)	0.643 (0.448, 0.839)
	OR (CI-95%-OR)	19.26(2.87-129.03)	1.9e+15(0.00-Inf)	39.99(1.13-1413.75)	1.9e+15(0.00-Inf)	47.37(0.74-3051.87)	1090062.58(0.00-Inf)	5.82(1.60-21.22)	23.46(1.06-516.83)	9.8e+41(0.00-Inf)	42485.33(0.00-Inf)	0.83(0.61-1.13)
	OR p-value	5.72E-12	6.06E-09	2.87E-11	6.06E-09	4.62E-05	3.60E-07	5.50E-05	1.53E-05	7.68E-06	3.60E-07	0.176
	Kruskal test p-value	1.38E-08	1.12E-04	1.33E-07	1.12E-04	0.000675	3.11E-04	0.001	0.001	0.002	3.11E-04	0.001
PCA index score	OR (CI-95%-OR)	27.96(2.76-283.22)	0.84(0.087-8.01)	6.37(1.53-26.51)	0.99(0.28-3.57)	1.82(0.14-23.33)	16.07(0.67-384.22)	6.52(0.35-120.14)	3.25(0.21-50.31)	148.36(0.32-68194.78)	5.93(0.37-94.45)	34.75(0.41-2922.49)
	OR p-value	1.60E-05	0.876	0.002	0.990	0.647	0.053	0.173	0.385	0.036	0.186	0.012
	Kruskal test p-value	0.000131	0.426	0.004	0.520	0.322	0.191	0.233	0.477	0.030	0.251	0.003

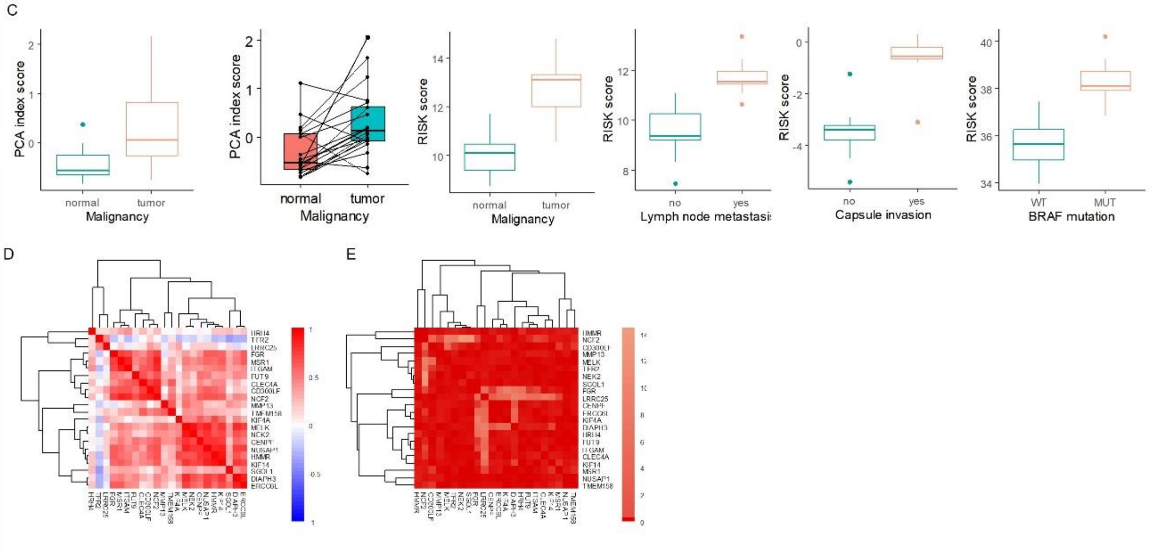

Supplementary Figure 17. 23-DEGs panel diagnostic and prognostication performance in the GSE29265 dataset. Dataset contains 20 PTCs, with 20 corresponding normal counterpart, and 9 ATCs. Clinical parameters shared only for PTC samples. **A.** Principal component analysis on thyroid samples according to 22 AC-DEGs (*PSG8* not included in the GPL570 Affymetrix platform), samples stratified according to the samples' malignancy (left) and histological type (middle), and 22 DEGs highly correlated (right). **B.** Diagnostic and prognostication performance of the 22-DEGs panel tested with the risk score ROC curve and logistic regression (top) and PCA index score linear regression analyses (bottom), in all samples (left), in the PTC samples with their normal counterparts (middle), and only in PTCs (right). Regression likelihood ratio p-value and Kruskal-Wallis p-value for corresponding distribution analysis were estimated because of the regression model convergence leading to OR calculation inaccuracy. p-value significant < 0.05. **C.** From left to right, boxplots representing the PCA-Index score distribution according to malignancy in all samples, and malignancy in PTCs with their normal counterparts, the 22-Risk score distribution according to malignancy in all samples, and lymph node metastasis, capsule invasion, and *BRAF*^{V600E} mutation parameters in PTC samples. **D.** Spearman correlation matrix of the 22 DEGs in the PTC cohort. **E.** heatmap representing the adjusted p-values comparing the GSE29265 22-DEG panel correlation matrix with the

© 2022. Ruiz E et al. Published online at http://www.aginganddisease.org/EN/10.14336/AD.2022.1021

SUPPLEMENTARY DATA

TCGA 22-DEG panel correlation matrix. Redder is the p-value and less the comparison is significant showing that the 22 DEGs behave similarly in the GSE29265 dataset as in the TCGA old cohort. ATC, Anaplastic thyroid carcinoma; CI-95%, 95% confidence interval; CPTC, classic (papillary) TC; FPTC, follicular-variant PTC; MUT, mutant; NPV, Negative predictive value; OR, Odd ratio; PPV, Positive predictive value; WT, wild-type.

A

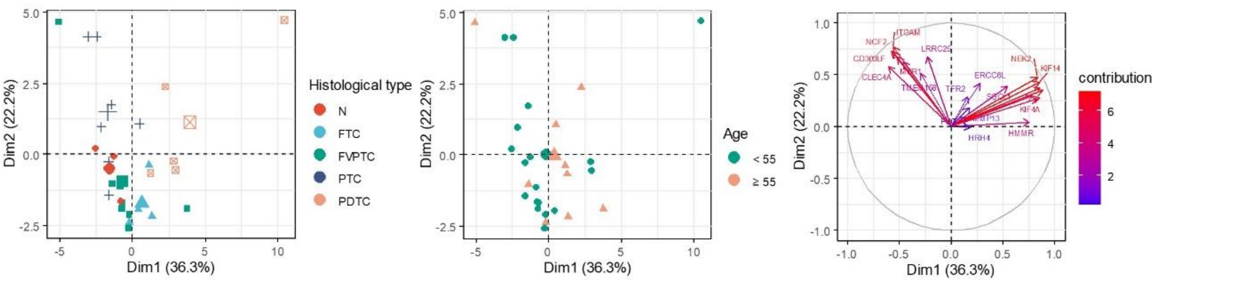

B

		All samples			Exclusion FTC		PTC		
		Malignancy (Tumors vs Normal)	Malignancy (PTC vs Normal)	Age (≥ 55 vs < 55)	Malignancy (Tumors vs Normal)	Age (≥ 55 vs < 55)	Histological type (CPTC/PDTC vs FPTC)	Histological type (PDTC vs F/CPTC)	Age (≥ 55 vs < 55)
RISK score	Cut-off	0.59	0.64	0.47	0.64	0.98	0.75	1.00	0.03
	Sensitivity	1.00	1.00	1.00	1.00	1.00	1.00	1.00	1.00
	Specificity	1.00	1.00	1.00	1.00	1.00	1.00	1.00	0.93
	PPV	1.00	1.00	1.00	1.00	1.00	1.00	1.00	0.86
	NPV	1.00	1.00	1.00	1.00	1.00	1.00	1.00	1.00
	AUC	1 (1-1)	1 (1-1)	1 (1-1)	1 (1-1)	1 (1-1)	1 (1-1)	1 (1-1)	0.988 (0.955-1.021)
	OR (CI-95%-OR)	8.9×10^{9} (0.00-Inf)	1.7×10^{13} (0.00-Inf)	2.1×10^{38} (0.00-Inf)	1.7×10^{13} (0.00-Inf)	4.3×10^{9} (0.00-Inf)	8.5×10^{94} (0.00-Inf)	619431.79 (0.00-Inf)	7.17 (1.02-50.10)
	OR p-value (likelihood ratio test)	1.42E-05	2.44E-05	4.55E-09	2.44E-05	2.77E-07	2.12E-07	2.11E-06	1.88E-05
	Kruskal test p-value	0.005	0.006	3.10E-05	0.006	3.56E-04	2.13E-04	0.001	7.21E-04
PCA index score	OR (CI-95%-OR)	4.62 (0.10-210.38)	6.69 (0.13-343.20)	1.27 (0.24-6.61)	6.31 (0.12-345.65)	2.13 (0.31-14.68)	97.77 (1.49-6435.29)	12.27 (0.70-215.68)	1.80 (0.24-13.69)
	OR p-value (likelihood ratio test)	0.365	0.267	0.781	0.296	0.441	0.004	0.040	0.569
	Kruskal test p-value	0.487	0.361	0.504	0.361	0.208	0.009	0.050	0.284

C

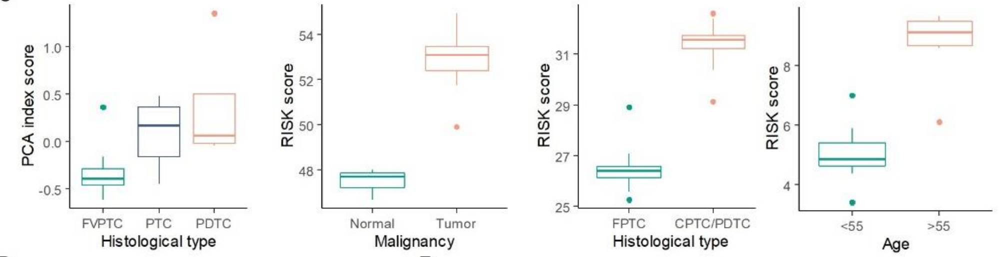

D E

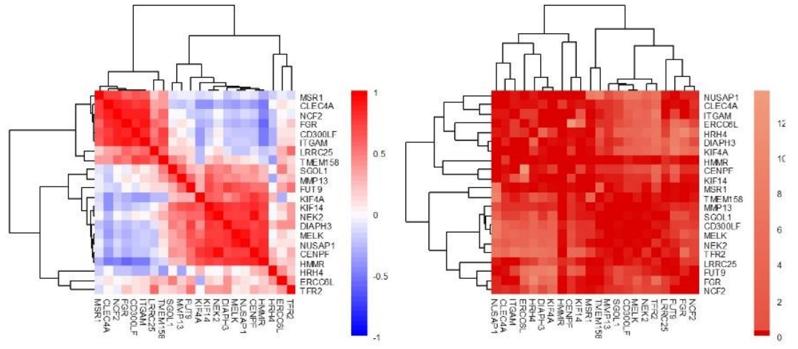

Supplementary Figure 18. 23-DEGs panel diagnostic and prognostication performance in the GSE53157 dataset. Dataset contains 27 samples (four FTCs, eight FPTC, five PDTC, seven classical PTC, and three normal samples). **A.** Principal component analysis on

© 2022. Ruiz E et al. Published online at http://www.aginganddisease.org/EN/10.14336/AD.2022.1021

thyroid samples according to 22 AC-DEGs (*PSG8* not included in the GPL570 Affymetrix platform), samples stratified according to the samples' histological (left) and age (middle), and 22 DEGs highly correlated (right). **B.** Diagnostic and prognostication performance of the 22-DEGs panel tested with the risk score ROC curve and logistic regression (top) and PCA index score linear regression analyses (bottom). Regression likelihood ratio p-value and Kruskal-Wallis p-value for corresponding distribution analysis were estimated because of the regression model convergence leading to OR calculation inaccuracy. p-value significant < 0.05. **C.** From left to right, boxplots representing the PCA-Index score distribution according to histological type in tumor samples, the 22-Risk score distribution according to malignancy in all samples, and histological type and age parameters in PTC samples. **D**. Spearman correlation matrix of the 22 DEGs in the PTC cohort. **E.** heatmap representing the adjusted p-values comparing the GSE53157 22-DEG panel correlation matrix with the TCGA 22-DEG panel correlation matrix. Redder is the p-value and less the comparison is significant showing that the 22 DEGs behave similarly in the GSE53157 dataset as in the TCGA old cohort. CI-95%, 95% confidence interval; CPTC, classic (papillary) TC; FPTC, follicular-variant PTC; MUT, mutant; NPV, Negative predictive value; OR, Odd ratio; PDTC, Poor differentiated TCs; PPV, Positive predictive value; WT, wild-type.

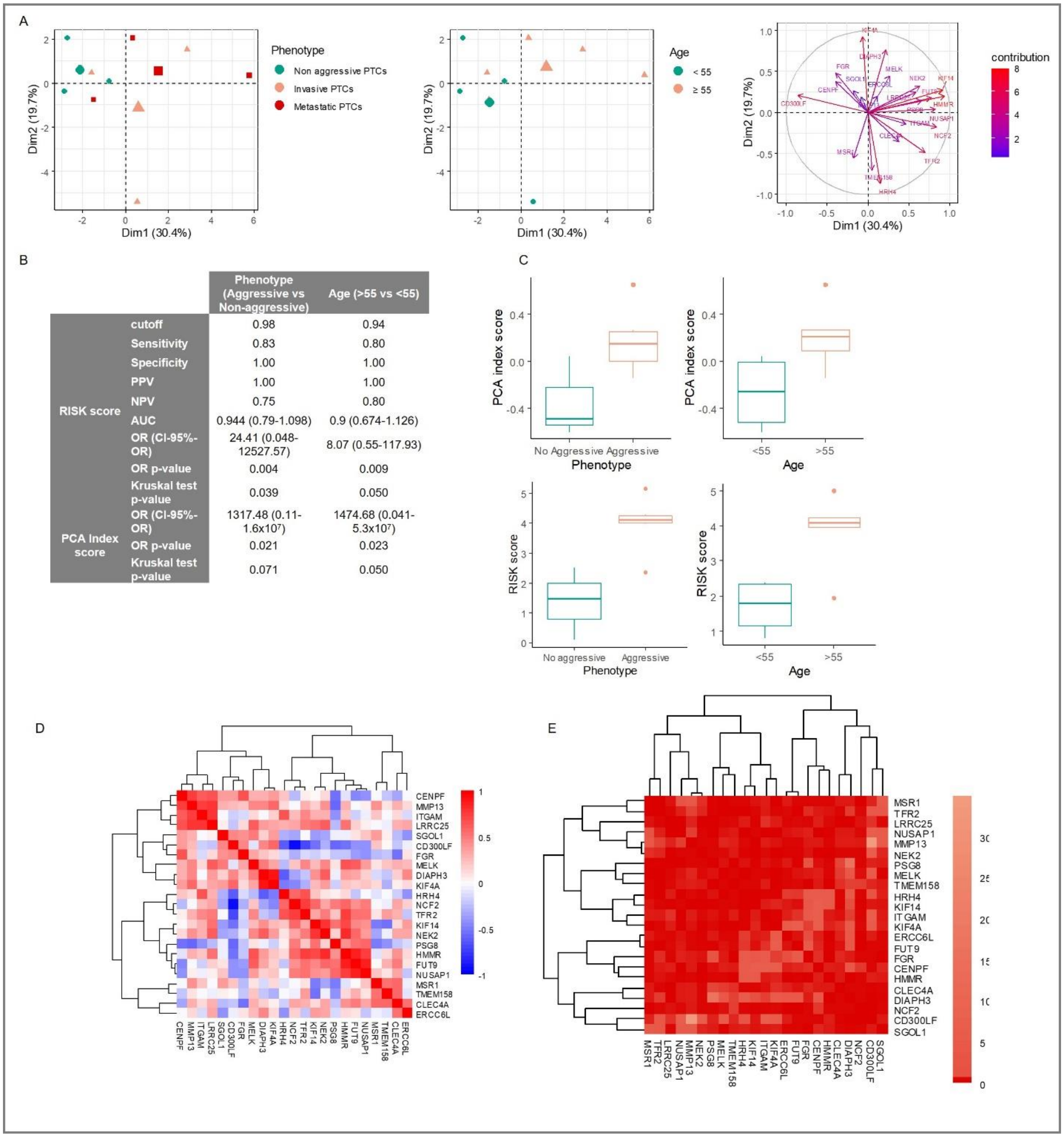

		Phenotype (Aggressive vs Non-aggressive)	Age (>55 vs <55)
RISK score	cutoff	0.98	0.94
	Sensitivity	0.83	0.80
	Specificity	1.00	1.00
	PPV	1.00	1.00
	NPV	0.75	0.80
	AUC	0.944 (0.79-1.098)	0.9 (0.674-1.126)
	OR (CI-95%-OR)	24.41 (0.048-12527.57)	8.07 (0.55-117.93)
	OR p-value	0.004	0.009
	Kruskal test p-value	0.039	0.050
PCA Index score	OR (CI-95%-OR)	1317.48 (0.11-$1.6x10^7$)	1474.68 (0.041-$5.3x10^7$)
	OR p-value	0.021	0.023
	Kruskal test p-value	0.071	0.050

© 2022. Ruiz E et al. Published online at http://www.aginganddisease.org/EN/10.14336/AD.2022.1021

SUPPLEMENTARY DATA

Supplementary Figure 19. 23-DEGs panel prognostication performance in the GSE129879 dataset. Dataset contains nine PTCs including three characterized as non-aggressive, three as invasive, and three as metastatic. **A.** Principal component analysis on thyroid samples according to 23 AC-DEGs, samples stratified according to the samples' phenotype (left) and age (middle), and 23 DEGs highly correlated (right). **B.** Prognostication performance of the 23-DEGs panel tested with the risk score ROC curve and logistic regression (top) and PCA index score linear regression analyses (bottom), for phenotype (left) and age (right) parameters. Regression likelihood ratio p-value and Kruskal-Wallis p-value for corresponding distribution analysis were estimated because of the regression model convergence leading to OR calculation inaccuracy. p-value significant < 0.05. **C.** Boxplots representing the 23-Risk score distribution according to phenotype (left) and age (right). **D.** Spearman correlation matrix of the 23 DEGs in the PTC cohort. **E.** heatmap representing the adjusted p-values comparing the GSE129879 23-DEG panel correlation matrix with the TCGA 23-DEG panel correlation matrix. Redder is the p-value and less the comparison is significant showing that the 23 DEGs behave similarly in the GSE129879 dataset as in the TCGA old cohort. CI-95%, 95% confidence interval; NPV, Negative predictive value; OR, Odd ratio; PPV, Positive predictive value.

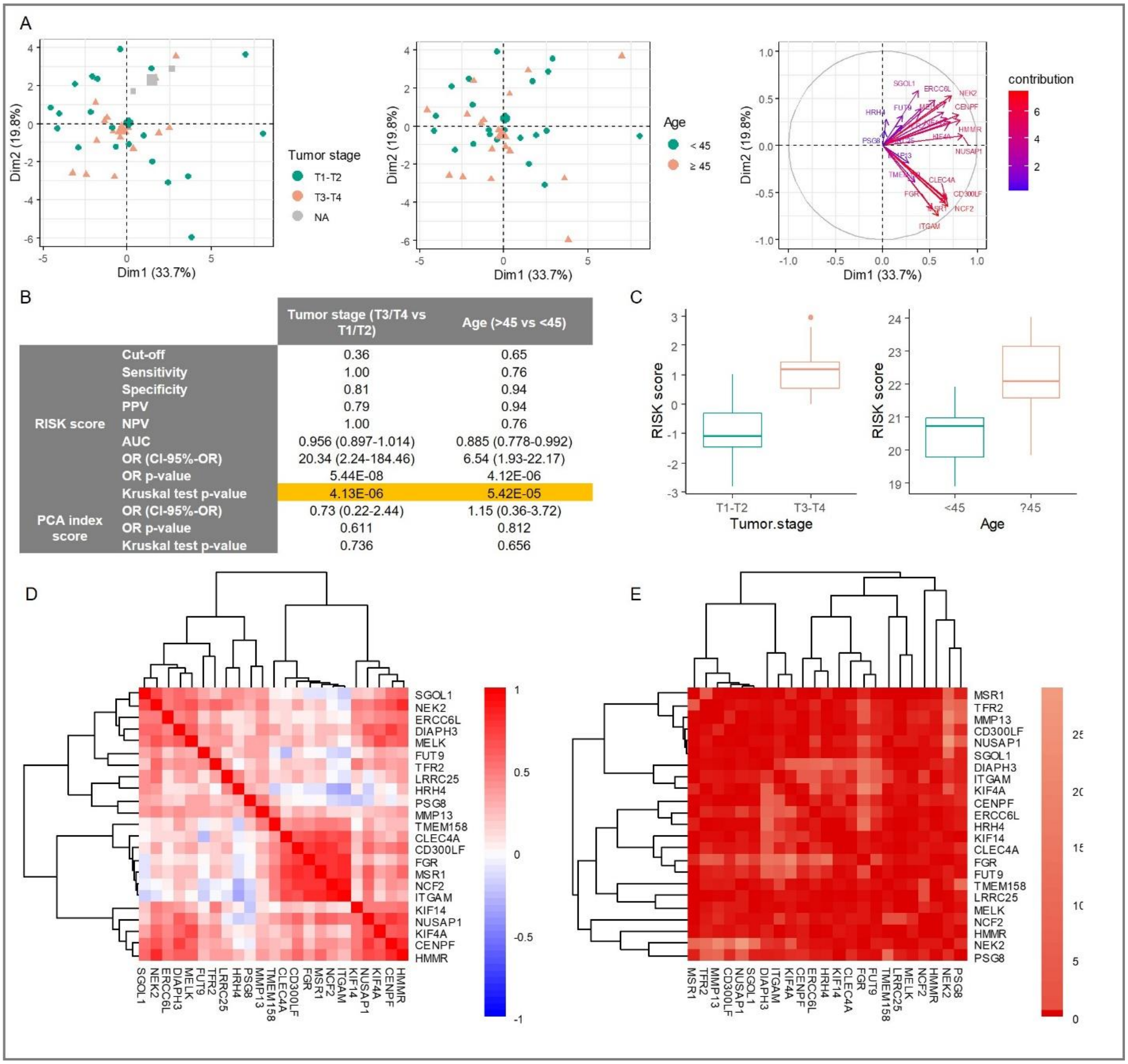

		Tumor stage (T3/T4 vs T1/T2)	Age (>45 vs <45)
RISK score	Cut-off	0.36	0.65
	Sensitivity	1.00	0.76
	Specificity	0.81	0.94
	PPV	0.79	0.94
	NPV	1.00	0.76
	AUC	0.956 (0.897-1.014)	0.885 (0.778-0.992)
	OR (CI-95%-OR)	20.34 (2.24-184.46)	6.54 (1.93-22.17)
	OR p-value	5.44E-08	4.12E-06
	Kruskal test p-value	4.13E-06	5.42E-05
PCA index score	OR (CI-95%-OR)	0.73 (0.22-2.44)	1.15 (0.36-3.72)
	OR p-value	0.611	0.812
	Kruskal test p-value	0.736	0.656

Supplementary Figure 20. 23-DEGs panel prognostication performance in the GSE65074 dataset. Dataset contains 38 PTCs including 21 and 15 characterized as T1/T2 and T3/T4, respectively. **A.** Principal component analysis on thyroid samples according to 23 AC-DEGs, samples stratified according to the samples' tumor stage (left) and age (middle), and 23 DEGs highly correlated (right). **B.** Prognostication performance of the 23-DEGs panel tested with the risk score ROC curve and logistic regression (top) and PCA index score linear regression analyses (bottom), for tumor stage (left) and age (right) parameters. Regression likelihood ratio p-value and

© 2022. Ruiz E et al. Published online at http://www.aginganddisease.org/EN/10.14336/AD.2022.1021

SUPPLEMENTARY DATA

Kruskal-Wallis p-value for corresponding distribution analysis were estimated because of the regression model convergence leading to OR calculation inaccuracy. p-value significant < 0.05. **C.** Boxplots representing the 23-Risk score distribution according to tumor stage (left) and age (right). **D.** Spearman correlation matrix of the 23 DEGs in the PTC cohort. **E.** heatmap representing the adjusted p-values comparing the GSE65074 23-DEG panel correlation matrix with the TCGA 23-DEG panel correlation matrix. Redder is the p-value and less the comparison is significant showing that the 23 DEGs behave similarly in the GSE65074 dataset as in the TCGA old cohort. CI-95%, 95% confidence interval; NPV, Negative predictive value; OR, Odd ratio; PPV, Positive predictive value.

© 2022. Ruiz E et al. Published online at http://www.aginganddisease.org/EN/10.14336/AD.2022.1021
